# Isolation and Identification of *Talaromyces* sp. Strain Q2 and Its Biocontrol Mechanisms Involved in the Control of *Fusarium* Wilt

**DOI:** 10.3389/fmicb.2021.724842

**Published:** 2021-10-08

**Authors:** Yehan Tian, Yang Zhao, Xuesong Fu, Chengming Yu, Kexiang Gao, Huixiang Liu

**Affiliations:** College of Plant Protection, Shandong Provincial Key Laboratory for Biology of Vegetable Diseases and Insect Pests, Shandong Agricultural University, Shandong, China

**Keywords:** *Fusarium oxysporum*, microbial community, *Talaromyces purpurogenus*, biocontrol mechanisms, gene function

## Abstract

*Fusarium* wilt is an important disease of many food crops and often causes serious damages to yield and food quality. Consequently, numerous studies mainly focused on exploring the control strategy for *Fusarium oxysporum* as well as the mechanism of interaction between the *F. oxysporum* and other beneficial soil microorganisms. In this study, we have screened and identified an efficient biocontrol strain from the soil with infection of *F. oxysporum* f. sp. *momordica* (referred to as Fom), *Talaromyces purpurogenus* Q2 (referred to as TpQ2), which could be effective to reduce relative abundance of the rhizospheric Fom, leading to a significant decrease of *Fusarium* wilt disease incidence in bitter gourd during the greenhouse and field trails. TpQ2 can reduce the relative abundance of rhizospheric Fom through inhibition of growth and development of Fom. During the co-cultivation of TpQ2 and Fom, we confirmed that TpQ2 could significantly suppress the growth and development of Fom through disturbing the normal hyphae shape and function of the cell walls of Fom *via* secreting cell wall–degrading enzymes and suppression of the expression of cell wall biosynthesis genes, such as *FomCFEM*. In the meantime, TpQ2 showed a strong negative correlation with *F. oxysporum* in soil and positive correlation with beneficial indigenous microorganisms that had significant negative correlation with *Fusarium* populations, such as *Streptomycetes, Lysobacter*, and *Sphingobium.* To summarize, TpQ2 has a good biocontrol efficacy on *Fusarium* wilt of bitter gourd. The biocontrol mechanisms of TpQ2 on *Fusarium* wilt are complex and diverse.

## Introduction

*Fusarium oxysporum* is a *common* saprophytic fungus that exists ubiquitously in *soil*, and some of them can cause vascular wilt disease in many food crops, leading to significant economic losses worldwide annually (Xiao et al., 2008; Arie, 2010; Huang and Cai, 2017; Kashiwa et al., 2017). *F. oxysporum* can grow saprophytically in plant debris to maintain its viability and survive in soil in the form of dormant chlamydospores for 10–15 years without the presence of its host plants (McKeen and Wensley, 1961; Kaur et al., 2010; Gordon, 2017; Huang and Cai, 2017). Once pathogenic *F. oxysporum* colonizes the soil through its diseased host plants, it is almost impossible to remove it from the soil (Dita et al., 2018; Srinivas et al., 2019). Continuous cultivation of a single crop in the same field is one of the major factors for the increased colonization and infection of *F. oxysporum* and the buildup of *F. oxysporum* population (Zhao et al., 2016; Shen et al., 2017). The invasion of the pathogenic *F. oxysporum* population can trigger drastic changes in microbiome compositions of soil, leading to a decline of disease suppression and soil quality due to an increase of pathogenic microbes, loss of microbial diversity, and microbial community function. But, soil microorganisms will not resign oneself to death; increased abundances of effective microorganisms, such as *Streptomycetes, Lysobacter, Sphingobium*, and *Dyadobacter*, build the first defense line against soil-borne pathogens in the suppressive soil of *Fusarium* wilt (Fu et al., 2017). Disease-suppressive soil provides the best examples of microbe-associated defense against the invasion of pathogens. Therefore, the establishment of an effective strategy to stimulate beneficial indigenous microorganisms and decrease the abundances of pathogenic *Fusarium* is critical for the successful management of *Fusarium* wilt disease.

Beneficial indigenous microorganisms in soil are an important source of agricultural biocontrol agents. To date, various biocontrol agents isolated from soil have been used to control *Fusarium* wilt disease under field condition, including *Trichoderma* spp., *Streptomyces* spp., and *Bacillus* spp. (Kim et al., 2011; Abbasi et al., 2019; Castillo et al., 2019; Jiang et al., 2019). The control efficacy of *Bacillus subtilis* B28 against *Fusarium* wilt of chickpea is over 44% by means of improving the plant growth parameters (Karimi et al., 2012). *Paenibacillus polymyxa* NSY50 can suppress the growth of *F. oxysporum* in the cucumber rhizosphere, with the biocontrol efficacy up to 60% (Shi et al., 2017). *Trichoderma asperellum* PRR2 working together with *Trichoderma* sp. NRCB3 can reduce the incidence of *Fusarium* wilt of banana by 47% based on the plant growth–promoting traits and the inhibition of spore germination and growth of *F. oxysporum* (Thangavelu and Gopi, 2015). Meanwhile, several studies have also suggested that biological agents can not only directly inhibit pathogen growth but also induce changes in beneficial indigenous microbiome with negative consequences on pathogen density. Tao found out that the *Bacillus amyloliquefaciens* W19 can stimulate indigenous soil *Pseudomonas* populations to enhance the soil resistance to *F. oxysporum* (Tao et al., 2020). Although biocontrol methods do not yield the same level of crop protection as the synthetic pesticide applications and the control results are often inconsistent, biocontrol methods using microorganisms will become an important measure of disease management in the field through continued study of the indigenous beneficial microorganisms in soil.

This study is mainly focused on screening soil biocontrol agent (s) to *F. oxysporum* from infected soil by *F. oxysporum* and exploring its biocontrol mechanisms. We set an assay using the bitter gourd (*Momordica charantia*)–infecting *F. oxysporum* f. sp. *momordica* (referred to as Fom thereafter, ACCC 39204) as a model pathogen. Fom was artificially inoculated to a bitter gourd experimental field, the bitter gourd was planted in this soil for consecutive 5 years, and the infected soil by *F. oxysporum* f. sp. *momordicae* was cultivated successfully. A new *Talaromyces* sp. strain Q2 (referred to as TpQ2) was isolated from the pathogenic soil of *Fusarium* wilt and showed a biological control potential against Fom. The biocontrol mechanism(s) of TpQ2 on *Fusarium* wilt of bitter gourd was studied using transcriptomics, microbial metagenomics, and other methods. The research result will help for the effective control of *Fusarium* wilt in food crops.

## Materials and Methods

### Source of Fungal Strain and Plant

*Fusarium oxysporum* f. sp. *momordicae* strain SG-15 (referred to as Fom) from the Agricultural Culture Collection of China (accession number ACCC39204) was originally isolated from a bitter gourd plant showing wilting symptoms. *F. graminearum*, *F. moniliforme*, *F. oxysporum* f. sp. *cucumarinum*, *F. oxysporum* f. sp. *niveum*, *Pyricularia oryza*, *Trichothecium roseum*, *Cryphonectria parasitica*, *Cytospora chrysosperma*, *Phytophthora parasitica*, and *Rhizoctonia solani* were obtained and maintained in our laboratory. Bitter gourd cv. Ruyu-41 was used in this study.

### Experimental Disease Nursery, Soil Collection, and Sample Preparation

The experimental disease nursery was established in 2015 at the experimental station, Shandong Agricultural University, Shandong Province, China. Soil samples were collected from the nursery from August to November 2019 for the analysis of the occurrence of exogenetic Fom and its control methods. From 2017 to 2019, the bitter gourd plants grown in this nursery showed 100% *Fusarium* wilt infection.

### Isolation of Biocontrol Agents From the Infected Soil by Fom

Each collected sample (10 g) was added to 100 ml of sterile distilled water and incubated on an orbital shaker for 30 min with 200 rpm shaking. An extract (0.2 ml) from a soil sample was placed on a potato dextrose agar (PDA) plate inside a Petri dish, and the plate was incubated at 28°C for 3–5 days. Colonies on the plate were transferred onto fresh PDA plates to establish bacterial or fungal cultures. More than 100 strains were obtained and used in the subsequent analyses. All the microbes isolated from the pathogenic soil of *Fusarium* wilt were screened for antagonistic fungi by culturing them again on the PDA plates. An agar disk with Fom was cut from a plate and placed 6 cm away from the agar disc with a microbial culture on the same PDA plate, and incubated at 28°C for 7 days. The radial growth of fungal mycelium was measured. Then, the percentage of growth inhibition was calculated as follows: I (%) = (1–Dn/Do) × 100, where I (%) represents the percentage inhibition of Fom mycelium growth, Dn represents the mean diameter of Fom colony in the presence of an isolated microbial strain, and Do represents the mean diameter of Fom colony in the absence of an isolated microbial strain. A total of 14 isolated microbial strains with strong inhibitory activities against Fom growth were listed in Supplementary Table 1.

### Morphological and Molecular Characterizations of *Talaromyces* sp. Strain Q2

*Talaromyces* sp. strain Q2 (referred to as TpQ2) was grown on three individual PDA plates inside Petri dishes (90 mm in diameter) at 25°C in the dark. After 2–5 days incubation, colony morphology, microscopic characteristics of conidiophores, and conidia of TpQ2 were examined as previously described (Visagie et al., 2015; Su and Niu, 2018). For further analyses, TpQ2 was grown on the Czapek agar (CA), Czapek yeast extract agar (CYA), Sabouraud maltose agar (SMA), cornmeal agar (CMA), Martin media (MM), Sabouraud sucrose agar (SSA), Sabouraud dextrose agar (SDA), or oatmeal agar (OA) for 7 days at 25°C. The colony morphology and microscopic characters of TpQ2 were then examined as the above method. Productions of β-1, 3-glucanase, and chitinase in TpQ2 grown on the agar plates supplemented with laminarin or colloidal chitin were determined using the clear zone method. The production of β-1, 3-glucanase and chitinase in the TpQ2 treated with the different substrates were also determined using the β-1, 3-glucanase enzyme The activity Determination Kit and the chitinase enzyme Activity Determination Kit method follow the manufacturer Solarbio Biotechnology, Beijing, China.

DNA extraction and TpQ2 mycelia were harvested from the surface of PDA plates, frozen in liquid nitrogen, and extracted for genomic DNA using the Fungal Genomic DNA Extraction Kit (BioTeKe Biotech Co., Ltd., Beijing, China). Primers (Supplementary Table 2) were used to identify TpQ2 (White et al., 1990; Glass and Donaldson, 1995; Wang and Wang, 2013). The genomic DNA of TpQ2 was sequenced using the whole genome *de novo* sequencing technology on the Illumina NovSeq platform and the Pacbio Sequel platform (Illumina, San Diego, CA, United States).

### Solid Fermentation of *Talaromyces purpurogenus* Q2

Wheat grain, corn straw, and humic acid were used as main materials in the solid fermentation medium. The orthogonal test table L_9_(3^4^) was used to determine the optimal formula for the solid-state fermentation medium for TpQ2 (Supplementary Table 3). After drying, culture media were crushed and adjusted individually to the spore concentration of 2 × 10^7^ cfu⋅g^–1^ before further use.

### *In vitro* and *in vivo* Antagonistic Effect Assays

To determine the ability of TpQ2 to inhibit the growth of Fom, *F. graminearum*, *F. moniliforme*, *F. oxysporum* f. sp. *cucumarinum*, *F. oxysporum* f. sp. *niveum*, *P. oryza*, *T. roseum*, *C. parasitica*, *C. chrysosperma*, *P. parasitica*, and *R. solani* were performed in *in vitro* antagonistic effect tests on PDA plates as described above. To determine the control effects of TpQ2 on Fom infection in the greenhouse and field trials, bitter gourd seedlings with 2–4 leaves were transplanted to the healthy soil without Fom inoculation (S-Fom) and the pathogenic soil with further Fom inoculation (S+Fom) (Wang et al., 2019). For the greenhouse trial and field trial, 15 g of inoculum containing 1 × 10^6^ conidia⋅g^–1^ of TpQ2 was mixed with 3 kg of soil for the treatments of S-Fom+TpQ2 and S+Fom+TpQ2, or with an equal amount of heat-inactivated (121°C, 25 min) inoculum of strain Q2 for S-Fom and S+Fom. 15 days (pot) or 30 days (field) later, the incidence and disease index of *Fusarium* wilt were recorded, and the control effect of TpQ2 on Fom infection was estimated as described (Chen et al., 2015).

### Analyses of the Mechanism of Inhibition of the Fom Growth System by *Talaromyces purpurogenus* Q2

To investigate how TpQ2 inhibits Fom growth, two treatments were conducted [i.e., incubation with Fom alone (Fom) or with TpQ2 and Fom (TpQ2+Fom)] with three biological replicates per treatment. The spores of TpQ2 and Fom were rinsed separately from their culture plates with sterile water and diluted to the final concentration of 4 × 10^7^ conidia⋅ml^–1^ and 2.5 × 10^7^ conidia⋅ml^–1^, respectively. The conidial suspension of Fom (0.8 ml) was mixed with 0 ml (T0), 0.05 ml (T1), 0.5 ml (T2), 1.0 ml (T3), 2.5 ml (T4), or 5.0 ml (T5) of TpQ2 conidial suspension; inoculated to 100 ml PDB; and then cultured at 28°C for 3–5 days on an orbital shaker with 180 rpm shaking. The growth of Fom with different treatments was recorded by measuring the number of the conidia of Fom. The culture was filtered through three layers of gauze, and the filtrates were centrifuged at 8,000 rpm for 10 min to get the conidia of Fom. Then, we performed propidium iodide (PI) staining to detect the cell vitality of Fom conidia according to the method of Gao et al. (2016). The level of N-acetylglucosamine in the supernatants was identified to reveal the damage of TpQ2 on the cell wall of Fom. The conidia of Fom was used to determine the content of malondialdehyde (MDA) and the intracellular triglyceride of cells to reveal the damage of TpQ2 on the membrane function of Fom. The contents of N-acetylglucosamine, intracellular triglyceride, and MDA were identified by enzyme linked immunosorbent assay (ELISA) kits (Jiangsu Jingmei Biotechnology, Jiangsu, China). Finally, transcriptome sequencing was applied to study the mechanism of inhibition of the Fom growth system by TpQ2 at the gene level.

The total RNA was extracted from fungal mycelium using Trizol Reagent (Invitrogen, Carlsbad, CA, United States) and then evaluated using the NanoDrop spectrophotometer. The sequencing libraries were generated using IlluminaTruSeq^TM^ RNA Sample Preparation Kit (Illumina, San Diego, CA, United States). Briefly, mRNA was purified from the total RNA using poly-T oligo-attached magnetic beads. Fragmentation was performed using divalent cations under an elevated temperature in an Illumina proprietary fragmentation buffer. First-strand cDNA was synthesized using random oligonucleotides and SuperScript II (Invitrogen, Carlsbad, CA, United States). cDNA synthesis was subsequently performed using DNA Polymerase I and RNase H. After adenylation of the 3′ ends of the DNA fragments, Illumina paired-end adapter oligonucleotides were ligated to prepare for hybridization. The library fragments were purified within an AMPure XP system (Beckman Coulter, Beverly, MA, United States) and sequenced by Shanghai Personal Biotechnology Co. (Shanghai, China). To determine gene expressions Fom, the reference genome index was built using the Bowtie2 (2.2.6) software and the filtered reads were mapped to the reference genome of *Fusarium oxysporum* f. sp. *cubense* race 1 (referred to as Foc1^1^) using the Tophat 2 (2.0.14) software based on improved Burrows–Wheeler transform (BWT) algorithm. The HTSeq (0.9.1) was used to determine the read counts of individually identified gene versus the expressions of these genes in the control libraries. FPKM (fragments per kilobase of exon model per million mapped reads) was used to standardize the expression levels, and DESeq (1.30.0) was used to analyze the differential expressions of genes. The cut-off value of *P* < 0.05 and at least 2-fold change were set as the threshold for differential expression.

To validate the transcriptome data, several genes were selected and analyzed for their expressions through quantitative reverse-transcription polymerase chain reaction (qRT-PCR). The primers used for qRT-PCR were listed in Supplementary Table 5. Quantitative PCR was performed using the SYBR Green qPCR kit on a Light Cycler^®^ 96 instrument (Roche). The expression level of *EF1α* was used as the internal control. The relative expression levels of the analyzed genes were calculated using the 2^–△^^△^^*CT*^ method (Tang et al., 2020).

### Identification of a CFEM Domain-Containing Protein in Fom

To investigate the sequence homology with gene-encoding proteins with a common in fungal extracellular membrane (CFEM) domain in various *Fusarium* species based on the phylogenetic analysis and the function of this CFEM domain-containing protein (FomCFEM) in Fom, we constructed *FomCFEM* deletion mutants by replacing the entire *FomCFEM* gene in Fom with the hygromycin B phosphotransferase gene (*hph*). Briefly, a 760-bp upstream and a 1,027-bp downstream sequence flanking the *FomCFEM* gene were PCR-amplified using primer pair CFEM-AF/R and CFEM-BF/R, respectively. The hygromycin-resistance gene was then amplified from pCB1003 vector using primer pair HYG-F/HY-R and YG-F/HYG-R. An equal amount of PCR products (20 μg total) was mixed and transformed into Fom protoplasts. The transformed protoplasts were grown on the TB3 selection medium supplemented with 100 μg/ml hygromycin B, and the transformants were identified through PCR using primer pair CFEM-F/R, CFEM-K1F/R, and CFEM-K2F/R, respectively.

To further confirm these transformants, genomic DNA was extracted from the mycelia of individual transformants and digested with *Pvu*I restriction enzyme (Tang et al., 2020). The digested DNA samples were analyzed through Southern blot assays using a DNA probe prepared using PCR with the primer pair tCFEM-F/R and the DIG High Prime DNA Labeling and Detection Starter kit I (Roche Diagnostics, Mannheim, Germany). The primers used in this study are listed in Supplementary Table 4.

For complementation assays, a 2.6-kb fragment bearing 1.1 kb full-length *FomCFEM* gene and its 1.5 kb promoter was PCR-amplified with the primer pair Ech1-CF/R, and cloned into the pFL2 vector through gap repair. This fusion plasmid was transformed into the Δ*FomCFEM* mutant protoplasts to produce complemented (Δ*FomCFEM*-*C*) transformants. The G418-resistant transformants were identified through PCR.

To determine the effect of *FomCFEM* on Fom growth, the wild-type Fom (WT Fom), its deletion mutants (Δ*FomCFEM*), and complemented mutants (*FomCFEM-C*) were cultured individually on the PDA plates at 28°C for 5 days. At 7 days post-incubation, the colony morphology and conidia of these strains were examined. For stress tolerance assay, individual strains were grown onto the CM plates supplemented with 0.2 g⋅L^–1^ Congo red (CR), 1 mol⋅L^–1^ sorbitol, 0.05% SDS, 0.7 mol⋅L^–1^ NaCl, 0.7 mol⋅L^–1^ KCl, or 5 mmol⋅L^–1^ H_2_O_2_ as previously described (Zhu et al., 2017; Tang et al., 2020). To determine conidial germination, a 50 μl conidial suspension (1 × 10^5^ conidia⋅ml^–1^) of each strain was spotted on hydrophobic coverslips and incubated at 28°C for 12 h, and then examined under a Nikon Eclipse 90i microscope (Nikon, Tokyo, Japan). Virulence assay was conducted using bitter gourd seedlings as described above.

### Analyses of Effects of *Talaromyces purpurogenus* Q2 on Soil Microecology and Abundance of *F. oxysporum*

To assess the effects of TpQ2 on soil microecology and the abundance of *F. oxysporum*, changes were analyzed on the soil microbial community, physicochemical property, and soil enzyme in the experimental disease nursery of *Fusarium* wilt after continuously applying TpQ2 for 2 years. For soil analysis, three different treatments (five biological copies per treatment) were used, including soil without Fom inoculation (S-Fom), soil with further Fom inoculation (S+Fom), and soil with further Fom and *Talaromyces* sp. strain Q2 inoculation (S+Fom+TpQ2). One kilogram soil sample was collected from the rhizosphere of bitter gourd plants per plot and then divided into three subsamples for the use of beneficial microorganism isolation, DNA extraction, and soil physicochemical property tests. Soil samples used for DNA extraction and physicochemical property tests were stored at −80°C and 4°C, respectively; the soil physicochemical properties, such as available nitrogen (AN, LY/T1228-2015), available phosphorus (AP, NY/T1121.7-2014), available potassium (AK, NY889-2004), soil organic carbon (SOC, NY1121.6-2006), pH value (NY1121.2-2006), electrical conductivity (EC, HJ 802-2016), available iron (AFe, NY/T890-2004), and available manganese (AMn, NY/T890-2004) were analyzed and evaluated following the method of the national standards of China. The activities of fluorescein diacetate (FDA) hydrolase, urease (UE), polyphenol oxidase (PPO), and acid phosphatase (ACP) were determined using various soil enzyme activity test kits by the manufacturer (Solarbio Biotechnology, Beijing, China). The number of *Fusarium* in bitter gourd rhizosphere was isolated on selective medium with soil dilution plate methods (Castaño et al., 2013), then using light microscope to identify the number of *F. oxysporum* based on conidia morphology of Fom. Metagenomic sequencing of soil microbiome was conducted at Personal Biotechnology Co., Ltd. (Shanghai, China).

To sequence metagenome, the total genomic DNA was isolated from individual soil samples (0.5 g per sample) using the FastDNA Spin kit (MP Biomedical, Santa Ana, CA, United States). The resulting DNA samples were checked using a NanoDrop spectrophotometer (Thermo Fisher Scientific, Waltham, MA, United States) and then used to construct metagenome shotgun sequencing libraries using the Illumina TruSeq Nano DNA LT Library Preparation Kit. The libraries were sequenced on the Illumina HiSeq X-ten platform. The raw sequencing reads were processed to obtain quality-filtered reads for further analyses followed by co-occurrence network analyses as reported previously (Zhang et al., 2018; Fu et al., 2021).

### Statistical Analysis

All results were expressed as mean values ± standard deviation (sd). Statistical analysis was performed in SPSS 22.0 software. One-way analysis of variance (ANOVA) with the Student–Newman–Keuls test was performed to determine the statistical significance. There was a preliminary study on the variations caused by environmental factors associated with the soil microbiota with multiple stepwise regression analysis.

## Results

### Identification and Isolation Biocontrol Agents From the Infected Soil by Fom

To identify microbes with antagonistic activity to Fom, over 100 microbes were first identified through screening, and then 14 of them were found with strong antagonistic activities (52–83%) to Fom, including 7 bacterial isolates, 1 *Penicillium* sp., 1 *Talaromyces* sp., and 5 *Trichoderma* sp. (Supplementary Table 6). During pot trials, four microbes (i.e., bacterial strains SK2 and SK6, TpQ2, and *Trichoderma* sp. strain M2) showed strong inhibitory effects (68–79%) on Fom. Further studies showed that the control efficacy of strain Q2 on *Fusarium* wilt of bitter gourd was stable in the field. The control efficacy of TpQ2 on *Fusarium* wilt of bitter gourd was about 63.4% during greenhouse trials and 60.2% during field trials (Table 1). Besides, the infection of Fom in bitter gourd seedlings was clearly delayed in the presence of TpQ2. In this study, TpQ2 was also found to inhibit the mycelial growth of other common fungal pathogens, such as *F. graminearum*, *F. moniliforme*, and *R. solani* (Supplementary Table 7).

**TABLE 1:** Biocontrol efficacy of TpQ2 on bitter gourd *Fusarium* wilt disease during pot and field trials.

**Treatment**	**Pot**			**Field**		
	**Incidence (%)**	**Disease index**	**Control efficacy (%)**	**Incidence (%)**	**Disease index**	**Control efficacy (%)**
S-Fom	/	/	/	6.94 ± 2.40c	2.31 ± 1.22c	/
S-Fom+TpQ2	/	/	/	2.78 ± 2.41c	0.93 ± 0.80c	/
S+Fom	95.70 ± 7.45*	72.07 ± 24.85*	/	45.56 ± 3.85a	34.44 ± 3.92a	/
S+Fom+Q2	63.20 ± 25.19	26.38 ± 13.87	63.40	27.78 ± 5.09b	13.7 ± 1.34b	60.22

*S-Fom: the healthy soil without *Fusarium* inoculation; S-Fom+Q2: the healthy soil without *Fusarium* inoculation followed by treatment with strain Q2; S+Fom: the pathogenic soil with *Fusarium* inoculation; S+Fom+Q2: the pathogenic soil with *Fusarium* inoculation followed by treatment with strain Q2; Data are mean ± SD.*

* represents significant differences at *P* < 0.05 based on the *T*-test. Different letters in the same column indicate significant difference at *P* < 0.05 level by Student–Newman–Keuls test. The below is same.

### Morphology, Phylogenetic Analysis, and Biocontrol Potential of *Talaromyces purpurogenus* Q2

**FIGURE 1:**
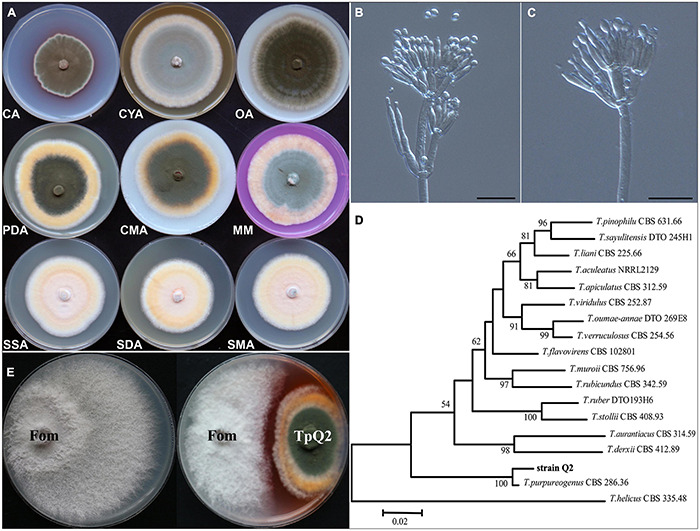
Morphologic and molecular identification of TpQ2 and its antagonistic effect on Fom growth. **(A)** Colonies of TpQ2 on different culture medium plates at 25°C after 7 day incubation. CA, Czapek agar; CYA, Czapek yeast extract agar; SMA, Sabouraud maltose agar; CMA, Corn meat agar; MM, Martin medium; SSA, Sabouraud sucrose agar; SDA, Sabouraud dextrose agar; OA, Oatmeal agar. **(B,C)** Morphology of TpQ2 conidiophore observed under a light microscopy. Scale bar = 20 μm. **(D)** The best-scoring Maximum Likelihood tree was constructed using the MEGA7.0 plateform and the combined data set containing partial internal transcribed spacer (*ITS*), beta-tubulin gene (*BenA*), and calmodulin gene (*CaM*) sequences. This phylogenetic tree shows the relationships of TpQ2 with other members in *Talaromyces*. The bootstrap support percentages are indicated at the nodes. The bootstrap support values at less than 50% are not shown. **(E)** Antagonistic effect of TpQ2 on Fom growth on culture medium plate.

Our observation result showed that TpQ2 colony grew moderately fast and reached 70.46, 49.44, 76.68, 76.22, 70.66, 75.32, 67.06, 61.00, and 66.56 mm in diameter on the PDA, CA, CYA, OA, CMA, MM, SSA, SDA, and SMA plates for 7 days, respectively (Figure 1A and Supplementary Table 8). Colonies of TpQ2 on the PDA plates were moderately deep, regular of edge, light yellow or white of hyphae of edge, grayish-green conidia pile, occasional exudates with reddish droplets, and dark ruby on the back of colony. Under the light microscope, TpQ2 hyphae appeared septate, smooth to coarse, and branched. Conidiophores produced by TpQ2 hyphal string were biverticillate with smooth-walled stipes and unswollen tops (Figures 1B,C), three-to-five metulae (8.0–13 μm × 2.3–3.0 μm), three-to-six acerose phialides (10–20 μm × 1.8–3.0 μm) per metulae, and smooth-walled oval or round conidia (3–3.5 μm × 2–2.5 μm). Conidia chains were loose and cylinder shaped and had no ascoma. The optimal growth temperature and pH value for TpQ2 were 30°C and 7.0, respectively, and the optimal carbon source was glucose (Supplementary Figure 1).

DNA sequencing results showed that the amplified fragment length of internal transcribed spacer (*ITS*, GenBank accession number KX432212), *beta-tubulin* gene (*BenA*, KY047419), and *calmodulin* gene (*CaM*, KX781300) of TpQ2 were 585 bp, 446 bp, and 705 bp long, respectively. The phylogenetic analysis results showed that TpQ2 was closely related to the type strain *T. purpureogenus* CBS 286.36 (Figure 1D). Together with the morphological characteristics, the TpQ2 was identified as *T. purpureogenus.* TpQ2 showed strong inhibitory activities against the mycelium growth of Fom in the plate (Figure 1E). The genome of TpQ2 was assembled using the PacBio technology and gave 57 scaffolds (27.73 Mb) containing 9,600 genes with an average length of 1,633 bp (Supplementary Table 9). A total of 8,968 genes were annotated using the KOG platform, and 41.1% of them were found to have unknown functions (Supplementary Figure 2A). A total of 463 carbohydrate-active enzymes (CAZymes) were identified, including 200 glycoside hydrolases (GHs), 83 glycosyl transferases (GTs), 84 carbohydrate esterases (CEs), 79 auxiliary activities (AAs), 15 carbohydrate-binding modules (CBMs), and 2 polysaccharide lyases (PLs) (Supplementary Figure 2B). The numbers of chitinase (GH18), β-glucosidase (GH3), xylanase (GH16), and α-N-acetylgalactosidase (GH109) were significantly greater than that of other CAZymes (Supplementary Figure 2C). We analyzed the activities of β-1, 3-glucanase and chitinase of TpQ2 (Supplementary Figures 2D-I). The result showed that β-1, 3-glucanase and chitinase demonstrate different activities in different substrates. The β-1, 3-glucanase activity in culture filtrate from the TpQ2+Fom, TpQ2, and Fom was 3.40 U⋅ml^–1^, 2.51 U⋅ml^–1^, and 3.66 U⋅ml^–1^, respectively (Supplementary Figure 2E). The β-1, 3-glucanase activity in TpQ2 isolated from CW (the filamentous fragments of Fom), Lam (laminarin), or CW+Lam culture filtrate was higher than control treatment (CK) during the sampling period (Supplementary Figure 2F). The chitinase activity in culture filtrate from the TpQ2+Fom, TpQ2, and Fom was 1.97 U⋅ml^–1^, 1.79 U⋅ml^–1^, and 1.78 U⋅ml^–1^, respectively (Supplementary Figure 2H). The chitinase activity in TpQ2 isolated from CW, cChi (colloidal chitin), or CW+cChi culture filtrate was higher than control treatment (CK) during the sampling period (Supplementary Figure 2I). For induction of β-1, 3-glucanase or chitinase, the filamentous fragment of Fom was better than laminarin or colloidal chitin in the early stage of the sampling period, while a compound substrate was better than a single one. These results imply that the growth of Fom was inhibited by TpQ2 when the TpQ2 and Fom co-exist.

### *Talaromyces purpurogenus* Q2 Inhibited the Growth and Development of Fom During Co-cultivation

**FIGURE 2:**
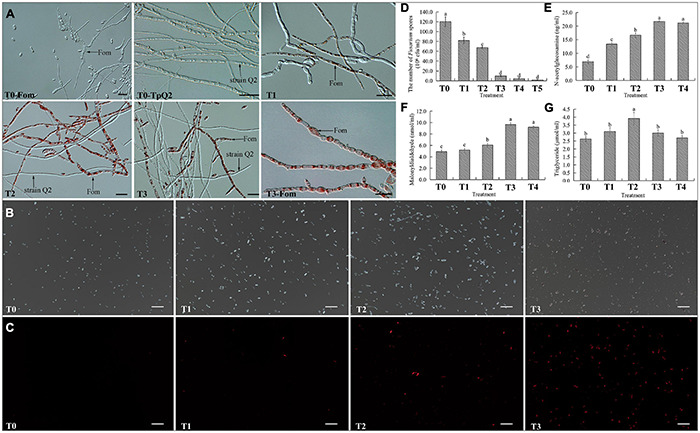
Effects of TpQ2 on the growth and reproduction of Fom. **(A)** Micrographs showing Fom and TpQ2 hyphal morphology. **(B,C)** Determination of vitality of Fom conidia in the T1–T4 growth media after propidium iodide (PI) staining. **(D)** Numbers of Fom spores at 5 days post co-culture of TpQ2 and Fom. **(E)** Contents of N-acetylglucosamine in the liquid co-culture supernatant. **(F)** Contents of malondialdehyde from Fom spores produced during co-culture of TpQ2 and Fom. **(G)** Contents of triglyceride from Fom spores produced during co-culture of TpQ2 and Fom. Arrows indicate Fom and TpQ2 hyphae. T0-Fom, Fom was grown in a medium in the absence of TpQ2. T0–TpQ2, TpQ2 was grown in a medium in the absence of Fom. T1–T5, The ratios of TpQ2 and Fom spores were 1:10, 1:1, 2:1, 5:1, and 10:1, respectively. Scale bars = 20 μm. Different letters in the same column indicate significant statistical difference at *P* < 0.05 level determined using the Student-Newman-Keuls test, the below is same.

To investigate whether TpQ2 could inhibit the growth and development of Fom and how TpQ2 inhibits Fom growth and development under natural conditions, TpQ2 and Fom in the liquid culture medium were first co-cultured. The result showed that the growth of TpQ2 and Fom in the pure culture and co-culture conditions were significantly different at 48 h. The Fom grown in the liquid medium appeared beige, thicker, and hazy, while the TpQ2 appeared lighter and then brownish 2 days later. After 2 days co-cultivation of TpQ2 and FOM (2:1, v/v; or greater), the culture broth turned red, and the TpQ2 mycelia became the dominant mycelia in the culture (Supplementary Figure 3). The hyphal morphology of Fom from the S-Fom samples was unchanged under the light microscope and grew and developed normally (Figure 2A). The hyphae of TpQ2 from all the samples also grew normally, and the color remained unchanged. However, the hyphae of Fom from T1–T3 samples became swollen and septa digestion, cytoplasm concentration, and appearance of globular materials, especially in T3 treatment (T3-Fom) (Figure 2A). The PI-stained Fom conidia showed more extensive red fluorescence in the presence of TpQ2 (T1–T4 samples) than that of control (T0) (Figures 2B,C), which indicated that TpQ2 caused cell membrane injury and vitality depression of Fom conidia. The numbers of Fom conidia from the T1, T2, T3, T4, and T5 liquid cultures were only 8.20 × 10^7^, 6.73 × 10^7^, 9.83 × 10^6^, 4.33 × 10^6^, and 1.77 × 10^6^ conidia⋅ml^–1^, respectively, compared to 1.20 × 10^8^ conidia⋅ml^–1^ conidia in the T0 (treatment with pure culture of Fom) treatment at the 5th-day post cultivation (Figure 2D). These results indicated that Fom hyphae growth and spore production were inhibited during the co-cultivation with TpQ2. The presence of red pigment in Fom cells indicated that the integrity of Fom cell wall and cell membrane was damaged by the presence of TpQ2. To validate this conclusion, we analyzed the effect of TpQ2 on the contents of MDA and triglyceride of Fom cells. The results showed that the contents of MDA and triglyceride of Fom cells in the T1, T2, T3, T4, and T5 were increased compared to T0 (Figures 2F,G). It is noteworthy that the content of N-acetylglucosamine in the liquid culture medium was gradually increased as the concentration of TpQ2 was increased (Figure 2E).

### Influence of *Talaromyces purpurogenus* Q2 on the Transcriptome of Fom During Co-cultivation

Over 336.47 million highly quality reads were obtained from the six libraries. For Fom libraries, 89.20-89.73% of the clean reads were mapped to the orthologous genome of Fom (the genome of Foc1). In the TpQ2+Fom libraries, 28.25-47.17% of the total reads were mapped to the orthologous genome of Fom (Supplementary Table 10). Genes that expressed differentially (DEGs) between the Fom and the Q2+Fom treatments were identified through comparison of the read counts normalized using the DEseq package. Through this comparison, a total of 4,893 DEGs were identified. These DEGs included 2,435 upregulated and 2,458 downregulated genes (Supplementary Figure 4A). The Gene Ontology (GO) and KEGG analyses showed that these DEGs could be classified into the categories of biological process (BP), molecular functions (MFs), and cellular component (CC) (Supplementary Figures 4B,C). For the TpQ2+Fom treatment, most genes associated with membrane and transmembrane transport were significantly enriched. To identify the biochemical pathways that regulate these DEGs during the inhibition of TpQ2 on Fom growth, these DEGs to the Kyoto Encyclopedia of Genes and Genomes (KEGG) database were mapped. The result showed that these DEGs could be classified into the pathways of metabolism, genetic information processing, environmental information processing, and cellular processes. The top 20 KEGG pathways affected by TpQ2 were shown in Supplementary Figure 4. The result further indicated that TpQ2 could significantly influence carbohydrate metabolism, amino acid metabolism, lipid metabolism, energy metabolism, the component of membrane, and membrane transport in Fom.

**FIGURE 3:**
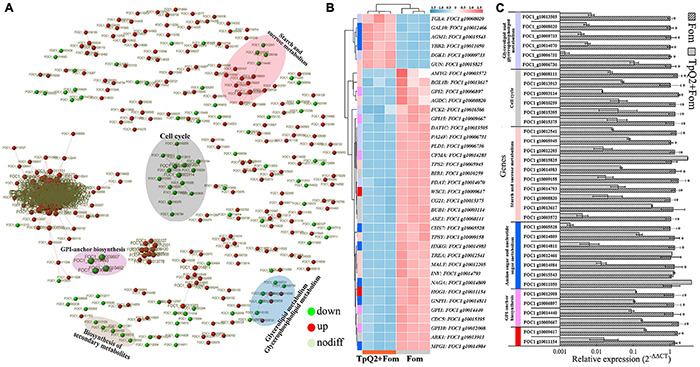
Effects of TpQ2 on the metabolic pathways and gene expressions in Fom during co-culture. **(A)** Protein-protein interaction networks were constructed using by 371 differentially expressed genes (DEGs) identified in Fom based on the STRING database. **(B)** Expression patterns of Fom DEGs determined through RNA sequencing. **(C)** Quantitative RT-PCR analyses of relative expressions of selected genes. *A statistically significant difference (Student’s *t*-test, *P* < 0.05) between the two treatments.

Based on the STRING output, the interactions of 371 genes were presented (Figure 3A). These genes were cell cycle related, starch and sucrose metabolism related, amino sugar and nucleotide sugar metabolism related, glycan biosynthesis and metabolism related, and biosynthesis of secondary metabolites related, respectively (Supplementary Table 11). Real-time PCR was then used to validate the expressions of 13 selected DEGs (Figures 3B,C), and the result showed that FOC1 g10015395 (CDC5), FOC1 g10015375 (CG21), FOC1 g10014984 (MPG1), FOC1 g10014983 (HXKG), FOC1 g10014811 (GNPI1), FOC1 g10014809 (NAGA), FOC1 g10014793 (INV), FOC1 g10014440 (GPI1), FOC1 g10013913 (ARK1), FOC1 g10013617 (BGL1B), FOC1 g10012541 (TREA), FOC1 g10012205 (MALT), FOC1 g10012008 (GPI18), FOC1 g10010259 (BIR1), FOC1 g10009667 (GPI15), FOC1 g10009617 (WSC1), FOC1 g10009158 (TPSY), FOC1 g10008820 (AGDC), FOC1 g10008111 (ASE1), FOC1 g10006897 (GPI2), FOC1 g10005945 (TPS2), FOC1 g10005528 (CHS7), FOC1 g10003572 (AMYG), and FOC1 g10003114 (BUB1) were down-regulated in the TpQ2+Fom samples. FOC1 g10015825 (GUN), FOC1 g10015543 (AGM1), FOC1 g10012466 (GAL10), and FOC1 g10011050 (YBB2) were upregulated in the TpQ2+Fom samples. Four GPI biosynthesis genes and one chitin synthase gene were downregulated in the TpQ2+Fom samples. These results indicated that TpQ2 could inhibit the growth of Fom by disturbing the cell wall biosynthesis or cell membrane permeability of Fom.

### *FomCFEM* Is an Important Factor for Cell Wall Stress and Virulence of Fom

**FIGURE 4:**
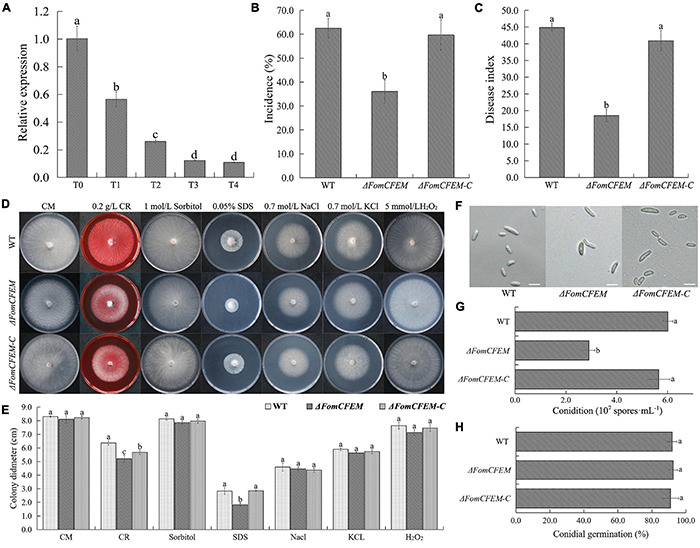
Effect of TpQ2 on the expression of a protein with a CFEM domain (*FomCFEM*) in Fom and its function. *FomCFEM* contains a CFEM (common in fungal extracellular membrane) domain, which is conserved among some fungal extracellular membrane proteins and has eight characteristic cysteine residues in its N terminal region. **(A)** Effect of TpQ2 on the expression of *FomCFEM* during co-culture of TpQ2 and Fom. **(B,C)** Pathogenicity of the wild type Fom, Δ*FomCFEM* deletion mutant, and Δ*FomCFEM-C* complementation mutant in bitter guard plants. **(D,E)** Morphology and diameter of the of wild type Fom, Δ*FomCFEM* deletion mutant, and Δ*FomCFEM-C* complementation mutant on the CM, CM+0.2 g⋅L^−1^ Congo red, CM+1 mol⋅L^−1^ sorbital, CM+0.05% SDS, CM+0.7 mol⋅L^−1^ NaCl, CM+0.7 mol⋅L^−1^ KCl, and CM+5 mmol⋅L^−1^ H_2_O_2_ medium. Changes of colony morphology were used to show the sensitivities of the wild type Fom, Δ*FomCFEM* deletion mutant, and Δ*FomCFEM-C* complementation mutant to osmotic, cell wall-damaged and oxidative stresses. **(F)** Morphology of conidia of the wild type Fom, Δ*FomCFEM* deletion mutant, and Δ*FomCFEM-C* complementation mutant. **(G)** Conidiation of the wild type Fom, Δ*FomCFEM* deletion mutant, and Δ*FomCFEM-C* complementation mutant. Conidiation was determined after 5 days culture in 100 ml PDA culture medium. **(H)** Spore germination of the wild type, Δ*FomCFEM* deletion mutant and Δ*FomCFEM-C* mutant. Spore germination rates were determined at 12 h after culture at 25°C. Different letters in the same column indicate significant statistical difference at *P* < 0.05 level determined using the Student-Newman-Keuls test, the below is same.

In this study, a DEG gene (FOC1 g10014283) with a CFEM domain, a signal peptide, and a putative Glycosylphosphatidylinositol (GPI)–anchored site (amino acid N^291^ and A^292^) (Supplementary Figures 5A, 6) were selected and analyzed. Based on the sequence of FOC1 g10014283, the *FomCFEM* was cloned from *F. oxysporum* f. sp. *momordicae* strain SG-15. Phylogenetic cluster analysis by the minimum evolution algorithm indicated that the sequence with a CFEM domain was well-conserved in *F. oxysporum* (Supplementary Figure 5B). We then generated Δ*FomCFEM* deletion mutant and Δ*FomCFEM-C* complemented mutant plasmids, and transformed them separately into Fom (Supplementary Figures 5C, 7B). One Δ*FomCFEM* deletion mutant and one Δ*FomCFEM-C* complemented mutant were selected for further assays (Supplementary Figure 5D). Further analysis of the gene (*FomCFEM*) showed that its expression was significantly decreased in the TpQ2+Fom samples compared to the Fom samples (Figure 4A and Supplementary Table 11).

To investigate the role of *FomCFEM* in Fom pathogenicity, the rhizosphere of bitter gourd seedlings was inoculated with a conidial suspension (1 × 10^6^ spores⋅g^–1^ soil) from the WT, Δ*FomCFEM* mutant or Δ*FomCFEM-C* complemented mutant. The result showed that the incidence and disease index of bitter gourd treated with the Δ*FomCFEM* mutant were significantly decreased (Figures 4B,C), and the virulence of Δ*FomCFEM* mutant was reduced by 58.8% compared to the WT strain, indicating that *FomCFEM* is crucial for the virulence of Fom. Although the conidial production by Δ*FomCFEM* was also decreased compared to the WT and the Δ*FomCFEM*-*C* mutant strain, the conidium morphology and germination of these three strains were similar (Figures 4F-H). To investigate the role of *FomCFEM* in Fom stress tolerance, we inoculated these three strains to the CM medium supplemented with 0.7 mol⋅L^–1^ NaCl and 0.7 mol⋅L^–1^ KCl (for salt stress tolerance), 1 mol⋅L^–1^ sorbitol (osmotic stress tolerance), 5 mmol⋅L^–1^ H_2_O_2_ and 0.05% SDS, and 0.2 g⋅ml^–1^ CR (cell wall stress tolerance), respectively. The result showed that the Δ*FomCFEM* mutant was more sensitive to the cell wall stress compared to the WT and the Δ*FomCFEM-C* mutant (Figures 4D,E).

### Influence of *Talaromyces purpurogenus* Q2 on the Relative Abundance of *Fusarium oxysporum* in Soil

The relative abundance of *F. oxysporum* in the S+Fom+TpQ2 soil samples was not decreased significantly (Supplementary Figure 8A), but the colony-forming units (cfu) of *F. oxysporum* from the S+Fom+TpQ2 soil samples were decreased significantly on the Komada’s medium (Supplementary Figure 8B). Among the fungal species, *Talaromyces* was the largest taxonomic group in the S+Fom+TpQ2 soil samples compared to the S-Fom and S+Fom soil samples. The metagenomic sequencing result showed that the S+Fom+TpQ2 soil samples had more TpQ2 (2828.01 reads) than the S+Fom soil sample (51.87 reads) and the S-Fom soil samples (27.09 reads) (Supplementary Figures 8C,I). Although TpQ2 was able to colonize in soil, its relative abundance was gradually decreased (Supplementary Figure 8D). Exogenous TpQ2 showed a strong negative correlation with the relative abundance of *F. oxysporum* in soil (*R* = 0.648, *P* = 0.043).

### Influence of *Talaromyces purpurogenus* Q2 on Soil Physicochemical Properties and Soil Enzymes

The change of soil physicochemical properties and soil enzymes after Fom and TpQ2 inoculation were analyzed and summarized. There were significant differences in soil physicochemical properties and soil enzymes among the groups S-Fom, S+Fom, and S+Fom+TpQ2 (Supplementary Table 12). Compared with S-Fom, the content of soil organic matter (SOC), pH value, and available iron (AFe) content were increased by 57.7%, 2.0%, and 27.7%, respectively, in the S+Fom soil samples, while the activities of soil FDA hydrolase and PPO were decreased by 47.5% and 17.4%, respectively. Compared with S+Fom, the activities of soil FDA hydrolase, the activities of soil PPO, the activities of soil UE, the activities of soil ACP, the value of EC (electrical conductivity), the content of AP (available phosphorus), and the content of AMn (available manganese) were increased by 177.1%, 34.2%, 21.0%, 84.4%, 31.5%, 10.0%, and 79.7%, respectively, in the S+Fom+TpQ2 soil samples, while the value of pH was decreased by 3.5%. Meanwhile, Spearman’s correlation analysis showed that TpQ2 had a stronger correlation with S-FDA, S-UE, S-ACP, S-PPO, EC, AN, AK, SOC, and AMn compared with Fom, and Fom and TpQ2 had strong positive correlations with SOC, AFe, and AP.

### Influence of *Talaromyces purpurogenus* Q2 on Microbial Community Diversity and Composition

The metagenomic sequencing data from the 15 libraries contained 34,279,350 contigs with 2,025,759 to 2,599,296 sequences per library (average 2,161,810.6 sequences from the S-Fom, 2,336,707.2 sequences from the S+Fom libraries, and 2,357,352.2 sequences from the S+Fom+TpQ2 libraries) and 33,514,586 scaffolds with 1,931,781 to 2,469,478 sequences per library (average 2,106,715 scaffolds from the S-Fom, 2,287,862.4 scaffolds from the S+Fom libraries, and 2308339.8 sequences from the S+Fom+TpQ2 libraries). Based on the metagenomic sequencing data, a total of 10,230 microbes with 88 phyla and 2,611 genera were identified. There were significant differences in microbial composition and microbial diversity among the groups S-Fom, S+Fom and S+Fom+TpQ2 (Supplementary Figure 8). As shown in Supplementary Figure 8E, the abundance and Chao1 index of the microbes found in the S+Fom samples were slightly higher than that found in the S-Fom samples. The S+Fom samples had significantly higher bacterial Simpson index and Shannon index than that in the S-Fom samples. The fungal Simpson index and Shannon index of the S+Fom samples were lower than that of the S-Fom samples. Although the abundance of the observed species and chao1 index in the S+Fom+TpQ2 soil samples were only slightly lower than that in the S+Fom soil samples, the fungal Simpson index and Shannon index in the S+Fom+TpQ2 soil samples were significantly lower than that in the S+Fom soil samples. In contrast, the S+Fom+TpQ2 soil samples had significantly higher bacterial Simpson index and Shannon index than that in the S+Fom soil samples.

Compared with S+Fom, the relative abundances of Actinobacteria (phylum), Firmicutes (phylum), Ascomycota (phylum), Chytridiomycota (phylum), *Streptomyces*, *Conexibacter*, *Sphingomonas*, *Gemmatirosa*, *Sphingobium*, *Nocardioides*, *Lysobacter*, *Talaromyces*, *Aspergillus*, and *Chaetomium* in the S+Fom+TpQ2 soil samples were increased, while the relative abundances of *Fusarium*, *Cordyceps*, *Colletotrichum, Anthracocystis*, *Trichoderma*, and *Diplodia* were decreased in the S+Fom+TpQ2 soil samples (Supplementary Figures 8F-I). Meanwhile, the random forests machine learning algorithm revealed that the majority of biomarker taxa showed high relative abundance in the S+Fom and S+Fom+TpQ2, for example, Ascomycota, Thaumarchaeota, Euglenida, Firmicutes, Chytridiomycota, Actinobacteria, Nitrospirae, Bacteroidetes, and Microsporidia (Supplementary Figure 8J). Spearman’s correlation analysis also showed that TpQ2 had a stronger correlation with the majority of biomarker taxa compared with Fom. Meanwhile, the majority of biomarker taxa, Fom, and TpQ2 had significant effects on microbial community diversity, such as Simpson index and Shannon index (Supplementary Table 13). TpQ2 was positively correlated with bacterial Simpson index and Shannon index and negatively correlated with fungal Simpson index and Shannon index; Fom was positively correlated with bacterial Simpson index and fungal observed species. These results indicated that application of exogenous TpQ2 had great influences on the structure of microbial communities, and may be more beneficial to the development of indigenous bacterial communities.

### Exploring the Potential Interactions Between *Talaromyces purpurogenus* Q2 and Soil Microorganisms

**FIGURE 5:**
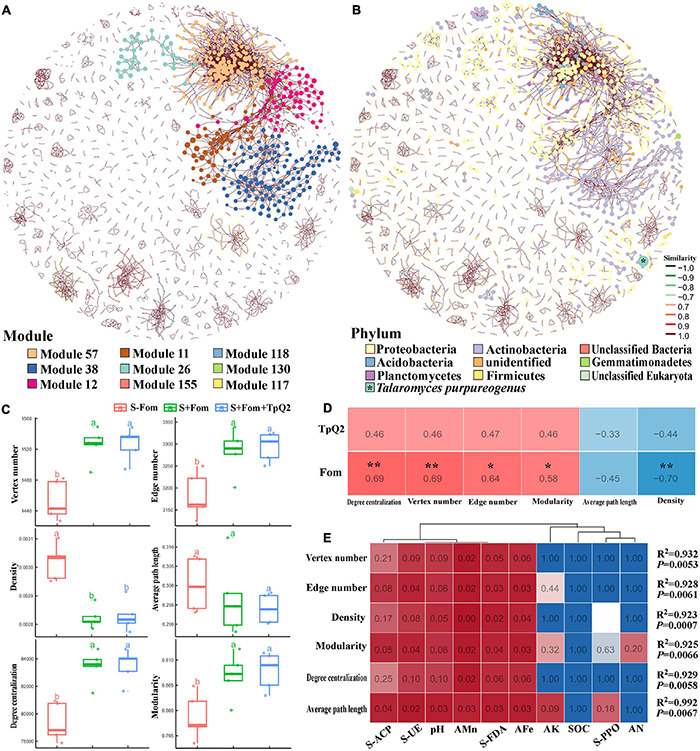
Co-occurrence networks of soil microbe communities constructed using the S-Fom, S+FOM, and S+Fom+TpQ2 data set and the correlation analysis. **(A)** Co-occurrence networks showing the module associations among soil microbes. **(B)** Compositions of the networks at the phylum level. **(C)** Key topological features of the microbial networks. **(D)** Spearman’s correlation analysis of the key topological features and Fom, TpQ2. **(E)** Multiple stepwise regression analysis of the environmental limiting factors of key topological features of the co-occurrence networks. Different letters in the same column indicate significant statistical difference at *P* < 0.05 level determined using the Tukey’s honestly significant difference (HSD) tests.

To explore co-occurrence patterns between TpQ2 and soil microorganisms, the co-occurrence networks from the S-Fom, S+Fom, and S+Fom+TpQ2 soil samples (Figures 5A,B) were constructed and analyzed. The degrees of the networks, the power-law distributions, and the average path length were significantly greater than that of the corresponding random networks, suggesting that the co-occurrence networks are a small world (Supplementary Figure 9A and Supplementary Table 14). The vertex number, edge number, modularity, and degree centralization were significantly higher for the sub-networks of the S+Fom+TpQ2 and S+Fom soil samples than that of the S-Fom soil samples, according to Tukey’s honestly significant difference (HSD) tests results, while the average path length and density were less for the sub-networks of the S+Fom+TpQ2 and S+Fom soil samples (Figure 5C). There was no difference in vertex number, edge number, modularity, degree centralization, average path length, and density between S+Fom and S+Fom+TpQ2 soil samples, suggesting that Fom might have a greater influence on key topological features of the microbial networks compared with TpQ2. Spearman’s correlation analysis also indicated that Fom had a stronger positive correlation with the key topological features of the microbial networks compared with TpQ2 (Figure 5D). Meanwhile, we found that FDA hydrolase, UE, ACP, soil pH, AFe, and AMn had a stronger influence on the network-level topological features than other assayed factors (Figure 5E and Supplementary Table 12). The co-occurrence network had 1,718 nodes (1,717 microbial nodes and 1 environmental node), 3,445 edges, and 72.73–74.80% microbial biomass (Figure 5 and Supplementary Table 14). Of these nodes, 98.72% of the nodes were peripherals, 1.05% of the nodes were connectors, and 0.17% of the nodes were module hubs, according to the distributions of soil microbial species determined using the Zi-Pi plot (Supplementary Figure 9B). Among the 1,717 main microbial species, 33.93% of them were Proteobacteria, 20.9% were Actinobacteria, 6.52% were Firmicutes, 3.78% were Thaumarchaeota, 3.08% were Euryarchaeota, and 2.91% were Ascomycota (Supplementary Figure 9C). The relative abundances of Actinobacteria and Firmicutes were higher in the S+Fom+TpQ2 soil sample than that in the S-Fom and S+Fom soil samples. These results indicated that there were potential interactions between TpQ2, Actinobacteria, and Firmicutes.

Then, the co-occurrence network was built using the information from the S+Fom and S+Fom+TpQ2 samples. The resulting co-occurrence network included 4,946 microbial and 8 environmental nodes, 16,275 edges, and 1,752 modules and accounted for 87.79–89.00% microbial biomass (Supplementary Figure 10 and Supplementary Table 14). In this co-occurrence network, TpQ2 was used as an environmental factor and showed positive correlations with Actinobacteria, Dictyoglomi, *K. flavida, F. casuarinae, Kitasatospora setae, Nocardiopsis alba, S. niveus*, and *Xylanimonas cellulosilytica* (Supplementary Figure 10E). In contrast, Fom showed negative correlation with Actinobacteria and Dictyoglomi.

## Discussion

Soil microbiota, including microbial biomass, microbial communities, microbial diversity, and abundances of specific microbial groups, is critical for the maintenance of soil health, quality, and enzyme activities (Bonilla et al., 2012; Zhao et al., 2016). When soil microorganisms are considered as soil genetic materials, each microbe can be considered as a functional gene, and these genes are associated and interacted with each other in soil environments. On the other hand, soil-borne pathogens can be considered as cancer genes that can significantly interrupt microbial networks, leading to changes of soil health. The invasion of soil by pathogenic microbes (i.e., *Ralstonia solanacearum* and *F. oxysporum*) can trigger drastic changes in microbiome compositions, leading to a decline of disease suppression and soil quality due to an increase of pathogenic microbes, loss of microbial diversity, and microbial community function (Shen et al., 2017; Wei et al., 2018). In the suppressive soil, microbe can defend the invasion of pathogens *via* the buildup of soil microorganisms as the first defense line against soil-borne pathogens. Chapelle found that Oxalobacteraceae, Burkholderiaceae, Sphingobacteriaceae, and Sphingomonadaceae are more abundant in the rhizosphere upon invasion of *R. solani* in the disease-suppressive soils (Chapelle et al., 2016). Through this study, we identified 14 biocontrol agents from the Fom-infected soil and these agents showed strong antagonistic activities to Fom growth on culture plates, including *Penicillium* sp., *Talaromyces* sp., *Trichoderma* sp., *Bacillus* sp., and *Streptomyces* sp. Comprehensive evaluation results showed that among these 14 biocontrol agents, *Talaromyces* sp. strain Q2 had more stable colonization in soil and control efficacy on *Fusarium* wilt in the greenhouse and field assays. Together with the morphological characteristics, we named strain Q2 as *T. purpureogenus* strain Q2 (TpQ2). Meanwhile, TpQ2 showed highly tolerant to a wide range of pH (4–10) and temperature (15°C–45°C) conditions, and biocontrol potential against multiple pathogenic fungi, especially Fom.

*Talaromyces* species are promising fungi and have many practical applications that are closely related to plants, animals, and humans, including the productions of enzymes and pigments. *Talaromyces* as a potential biological control agent (BCA) against fungal plant pathogen can be traced back to 1989. Spink and Rowe (1989) found out that *T. flavus* from rhizosphere soil had the potential to control *Verticillium dahliae* in potato. Kim et al. (2017) also found out that *T. flavus* isolated from ginseng seed showed antagonistic activities against fungal plant pathogens, such as *Rhizoctonia solani*, *Sclerotinia nivalis*, and *Phytophthora capsici*. Meanwhile, *Talaromyces* sp. can produce more CAZymes than sequenced *Penicillium* sp. based on the genome sequencing data (Mardones et al., 2018a, b; Varriale et al., 2018). This finding may reveal that *Talaromyces* sp. is a better BCA against plant fungal pathogens. To investigate the interaction between TpQ2 and Fom, we first conducted co-cultivation assays using TpQ2 and Fom. The result showed that the growth of Fom was significantly inhibited in the presence of TpQ2. In the presence of both TpQ2 and Fom, we found out that the production of β-1, 3-glucanase, and chitinase of TpQ2 was increased. Similar results were obtained when *Trichoderma viride*, *Aspergillus sterreus*, and *Leptosphaerulina* sp. were cultured together (Copete-Pertuz et al., 2019). Degradation of fungal cell walls by microbial chitinase, chitosanase, and β-1, 3-glucanase may be one of the explanations for the biocontrol of soil-borne plant pathogens (Borrero et al., 2004; Gherbawy et al., 2012; Ueki et al., 2017; Abbasi et al., 2019). In Ueki et al. (2017) reported that two strains of *Clostridium beijerinckii* produce cell wall–degrading enzymes and have the ability to inhibit *F. oxysporum* f. sp. *spinaciae*. On the other hand, our results obtained in this study also showed that the content of MDA and chitin in Fom mycelia were changed in the presence of TpQ2, indicating that the presence of TpQ2 altered of the membrane permeability and cell wall structure of Fom hyphae, and this alteration became more obvious after the density of TpQ2 was further increased. Transcriptome analysis results also indicated that Fom membrane components and transmembrane transport were seriously affected by the presence of TpQ2. This finding agreed with an earlier report showing that the membrane components and transmembrane transporter of *F. verticillioides* were affected by the presence of *Streptomyces* sp. strain AV05 (Strub et al., 2019).

Fungal cell wall and cell membrane are known to play important roles in cell shape, mating, and sporulation (Levin, 2011; Orlean, 2012). For fungal plant pathogen Fom, cell wall and cell membrane integrity not only play important roles in mycelium growth but also Fom and plant interactions (López-Fernández et al., 2013). In this study, we had discovered that several pathways associated with cell wall morphogenesis (i.e., starch and sucrose metabolisms, amino sugar and nucleotide sugar metabolisms, and GPI biosynthesis) were drastically influenced by TpQ2. The expressions of the cell wall and cell membrane–associated genes (i.e., *CHS1*, *CHS2*, *CHS3*, *CHS7*, *YEA4*, *GPI1*, *GPI2*, *GPI15*, *GPI12*, *SMP3*, and *GPI18*) were downregulated significantly under the stress of TpQ2. These associated genes with cell wall and cell membrane have been reported to play important roles in fungal growth and pathogenicity (Birkaya et al., 2009). GPI-anchored proteins, which are firmly bound to the cell wall by non-covalent interactions, are essential for the growth, viability, morphogenesis, signaling transmission, reproduction, and pathogenesis in all eukaryotic cells (Leidich et al., 1994; Pittet and Conzelmann, 2007; Li et al., 2018). GPI-anchored site is an essential component of the GPI-anchored proteins and has also been shown to be important for pathogenicity, osmotic stability, and mortality (Zhu et al., 2017). Once GPI biosynthesis is blocked, the growth and development of the fungus will be affected. In this study, we identified an FOC1 homologous gene from Fom, *FomCFEM*, with a putative GPI-anchored site. Our result showed that *FomCFEM* is an important regulator of virulence, growth, and stress tolerance of Fom. This result agrees with an earlier report showing that a gene with a CFEM domain and a putative GPI-anchored site is important in *B. cinerea* virulence, conidiation, and stress tolerance (Zhu et al., 2017). Therefore, we speculate that TpQ2 inhibits Fom growth through suppression of carbohydrate synthesis and cell wall biosynthesis of Fom. Biological control of *Fusarium* wilt disease has gained great interest in agriculture in recent years (Bubici et al., 2019), and many BCAs with various levels of effectiveness against Fom have now been reported (Xue et al., 2015; Fu et al., 2016; Wu et al., 2017), and most of them are isolated from soil. Meanwhile, it was reported that biocontrol bacteria or fungi start to sustain disease suppression after reaching threshold population sizes (Schlatter et al., 2017). In this study, TpQ2 started to suppress Fom growth and development when the population size of TpQ2 reached 1 × 10^6^ conidia⋅g^–1^, through human intervention. Meanwhile, the relative abundance of *Streptomyces*, *Sphingomonas*, *Gemmatirosa*, *Sphingobium*, *Nocardioides*, *Kribbella*, *Lysobacter*, *Mycobacterium*, *Micromonospora*, and *Chaetomium* in soil were increased in the process of biocontrol of *Fusarium* wilt by *T. purpurogenus* strain Q2.

Studies have reported that *Streptomycetes, Lysobacter*, and *Pseudomonas* display higher abundance in the disease-suppressive soil of *Rhizoctonia solani* and may be an important family member of antagonistic microorganisms (Postma et al., 2010). Disease incidence of *Fusarium* wilt was negatively correlated with *Sphingobium*, and the increased abundances of *Sphingobium* may be involved in disease suppression through taxa-specific suppression mechanisms (Fu et al., 2017). Balanced microbiota contributes to the effective biocontrol of soil-borne pathogenic diseases. The antagonism of soil probiotic microorganisms, such as Actinomycetes and Firmicutes strains, was the effective biological control of the guarantee of balanced microbiota on soil-borne pathogen. Although our results indicated that some beneficial indigenous microorganisms that had positive correlations with *T. purpureogenus* strain Q2 could be involved in the inhibition of the growth and development of Fom, there were no valid evidences to test and verify the result in the paper. A profound study needs to be made to reveal the interaction among Fom, TpQ2, and the microorganisms that have positive correlations with TpQ2 and to prove that TpQ2 could suppress the growth and development of Fom by impacting on the resident soil microbiome.

## Conclusion

*Talaromyces purpurogenus* Q2 isolated from the soil infested with the pathogen of *Fusarium* wilt was identified as a potential biocontrol agent, which showed strong antagonistic activity against multiple plant pathogens and a good control effect on *Fusarium* wilt of bitter gourd. The biocontrol mechanism of TpQ2 on *Fusarium* wilt was to reduce the relative abundance of rhizospheric Fom through inhibition of growth and development of Fom; the mechanism of TpQ2 inhibiting the growth and development of Fom may disturb the normal shape and function of the cell wall of Fom. Meanwhile, TpQ2 exhibited a strong negative correlation with *F. oxysporum* and a positive correlation with some resident soil microbes in soil. Those indigenous microorganisms showed significant negative correlation with *Fusarium*, such as Actinomycetes strains.

## Data Availability Statement

Additional data related to this study are available from the corresponding author upon reasonable request. All raw sequence data have been made available in the NCBI Sequence Read Archive (SRA) database under the accession number SRP. The raw Illumine sequence data of metagenomic data have been deposited in the sequence read archive (SRA submission: SUB9063041) at NCBI under Bioproject accession #PRJNA701786. The raw Illumine sequence data of RNA-seq are In review available (SRA accession: SRR10416247-SRR10416252) in the NCBI under Bioproject accession #PRJNA588316. The genome sequence data of Talaromyces purpureogenus strain Q2 is also available (Genomes submission: JABUIS000000000) in the NCBI under Bioproject accession #PRJNA635544.

## Author Contributions

YT and KG developed the concept of this study and are main contributors to writing the manuscript. YT, YZ, and XF performed all experiments, carried out the data analysis, and prepared the figures. YT, KG, CY, and HL contributed to the manuscript edit and review. All authors read and approved the final manuscript.

## Conflict of Interest

The authors declare that the research was conducted in the absence of any commercial or financial relationships that could be construed as a potential conflict of interest.

## Publisher’s Note

All claims expressed in this article are solely those of the authors and do not necessarily represent those of their affiliated organizations, or those of the publisher, the editors and the reviewers. Any product that may be evaluated in this article, or claim that may be made by its manufacturer, is not guaranteed or endorsed by the publisher.

## Footnotes

1
https://www.ncbi.nlm.nih.gov/genome/707?genome_assembly_id~=~202328

## Supplementary Material

Supplementary Figure 1 Influence of different nutrients and culture conditions on TpQ2 colony growth.

Supplementary Figure 2 Genome-wide identification and the potential function analysis of the beneficial TpQ2.

Supplementary Figure 3 Single and co-culture of TpQ2 and Fom.

Supplementary Figure 4 MA map analysis **(A)**, Gene Ontology analysis **(B)**, and KEGG enrichment analysis (C) using DEGs identified between the Fom and TpQ2+Fom treatments.

Supplementary Figure 5 Domains predicted in *FomCFEM*, phylogenetic tree, and PCR screen of Fom *FomCFEM* deletion and complementation mutants.

Supplementary Figure 6 Prediction of GPI site in *FomCFEM*.

Supplementary Figure 7 A schematic diagram showing detection of *FomCEFM* deletion mutants.

Supplementary Figure 8 Influences of TpQ2 on microbial alpha diversity index and community composition.

Supplementary Figure 9 Network level microbial features in the co-occurrence networks.

Supplementary Figure 10 Co-occurrence networks of soil microbial communities.

Supplementary Table 1 Disease incidence of *Fusarium* wilt of bitter gourd in field after transplantation for 45 days at 2015-2019.

Supplementary Table 2 The primers and type strains used in identification of strain TpQ2.

Supplementary Table 3 Solid fermentation formula of strain TpQ2.

Supplementary Table 4 The primers used in the function identification of *FomCFEM*.

Supplementary Table 5 The primer of DEGs used in the RT-qPCR.

Supplementary Table 6 The control efficacy of biocontrol agents on *Fusarium* wilt of bitter gourd.

Supplementary Table 7 Inhibition of strain TpQ2 against pathogenic fungi.

Supplementary Table 8 The colony diameter of strain TpQ2 on different medium.

Supplementary Table 9 The genomic information of strain TpQ2.

Supplementary Table 10 The data quality of RNA-seq.

Supplementary Table 11 The expression patterns of cell cycle and cell wall biosynthesis associated genes.

Supplementary Table 12 Response of soil chemical properties and soil enzyme activity to invasion of Fom and TpQ2.

Supplementary Table 13 Spearman’s correlation analysis to explain the correlation of key microorganisms, alpha diversity, and exogenous microorganisms.

Supplementary Table 14 Key topological features of soil microbial networks.

## References

[B1] Abbasi S. Safaie N. Sadeghi A. Shamsbakhsh M. (2019). *Streptomyces* strains induce resistance to *Fusarium oxysporum* f. sp. *lycopersici* race 3 in tomato through different molecular mechanisms. *Front. Microbiol.* 10:1505. 10.3389/fmicb.2019.01505 31333615 PMC6616268

[B2] Arie T. (2010). Phylogeny and phytopathogenicity mechanisms of soilborne *Fusarium oxysporum*. *J. Gen. Plant Pathol.* 76 403–405. 10.1007/s10327-010-0264-z

[B3] Birkaya B. Maddi A. Joshi J. Free S. J. Cullen P. J. (2009). Role of the cell wall integrity and filamentous growth mitogen-activated protein kinase pathways in cell wall remodeling during filamentous growth. *Eukaryot. Cell* 8 1118–1133. 10.1128/EC.00006-09 19502582 PMC2725558

[B4] Bonilla N. Gutiérrez-Barranquero J. A. Vicente A. D. Cazorla F. M. (2012). Enhancing soil quality and plant health through suppressive organic amendments. *Diversity* 4 475–491. 10.3390/d4040475

[B5] Borrero C. Trillas M. I. Ordovás J. Tello J. C. Avilés Manuel. (2004). Predictive factors for the suppression of *Fusarium* wilt of tomato in plant growth media. *Phytopathology* 94 1094–1101. 10.1094/PHYTO.2004.94.10.1094 18943798

[B6] Bubici G. Kaushal M. Prigigallo M. I. Gómez-Lama Cabanás C. Mercado-Blanco J. (2019). Biological control agents against *Fusarium wilt* of banana. *Front. Microbiol.* 10:616. 10.3389/fmicb.2019.00616 31024469 PMC6459961

[B7] Castaño R. Borrero C. Trillas M. I. Avilés M. (2013). Selection of biological control agents against tomato *Fusarium wilt* and evaluation in greenhouse conditions of two selected agents in three growing media. *J. Int. Organ. Biol. Control* 58 105–116. 10.1007/s10526-012-9465-z

[B8] Castillo A. G. Puig C. G. Cumagun C. (2019). Non-synergistic effect of *Trichoderma harzianum* and *Glomus* spp. in reducing infection of *Fusarium wilt* in banana. *Pathogens* 8:43. 10.3390/pathogens8020043 30935146 PMC6630906

[B9] Chapelle E. Mendes R. Bakker P. A. Raaijmakers J. M. (2016). Fungal invasion of the rhizosphere microbiome. *ISME J.* 10 265–268. 10.1038/ismej.2015.82 26023875 PMC4681858

[B10] Chen Z. D. Huang R. K. Li Q. Q. Wen J. L. Yuan G. Q. (2015). Development of pathogenicity and AFLP to characterize *Fusarium oxysporum* f. sp. momordicae isolates from bitter gourd in China. *J. Phytopathol.* 163 202–211. 10.1111/jph.12310

[B11] Copete-Pertuz L. S. Alandete-Novoa F. Placido J. Correa-Londono G. A. Mora-Martinez A. L. (2019). Enhancement of ligninolytic enzymes production and decolourising activity in *Leptosphaerulina* sp. by co-cultivation with *Trichoderma viride* and *Aspergillus terreus*. *Science of the Total Environment* 646 1536–1545. 10.1016/j.scitotenv.2018.07.387 30235638

[B12] Dita M. Barquero M. Heck D. Mizubuti E. Staver C. P. (2018). *Fusarium wilt* of banana: current knowledge on epidemiology and research needs toward sustainable disease management. *Front. Plant Sci.* 9:1468. 10.3389/fpls.2018.01468 30405651 PMC6202804

[B13] Fu L. Penton C. R. Ruan Y. Shen Z. Z. Xue C. Li R. (2017). Inducing the rhizosphere microbiome by biofertilizer application to suppress banana *Fusarium wilt* disease. *Soil Biol. Biochem.* 104 39–48. 10.1016/j.soilbio.2016.10.008

[B14] Fu L. Ruan Y. Tao C. Y. Li R. Shen Q. R. (2016). Continous application of bioorganic fertilizer induced resilient culturable bacteria community associated with banana *Fusarium wilt* suppression. *Sci. Rep.* 6:27731. 10.1038/srep27731 27306096 PMC4910074

[B15] Fu X. Ou Z. Zhang M. Meng Y. Li Y. Wen J. (2021). Indoor bacterial, fungal and viral species and functional genes in urban and rural schools in Shanxi Province, China-association with asthma, rhinitis and rhinoconjunctivitis in high school students. *Microbiome* 9:138. 10.1186/s40168-021-01091-0 34118964 PMC8199840

[B16] Gao T. Zhou H. Zhou W. Hu L. Chen J. Shi Z. (2016). The fungicidal activity of thymol against *Fusarium graminearum* via inducing lipid peroxidation and disrupting ergosterol biosynthesis. *Molecules* 21:770. 10.3390/molecules21060770 27322238 PMC6272974

[B17] Gherbawy Y. Elhariry H. Altalhi A. El-Deeb B. Khiralla G. (2012). Molecular screening of *Streptomyces* isolates for antifungal activity and family 19 chitinase enzymes. *J. Microbiol.* 50 459–468. 10.1007/s12275-012-2095-4 22752910

[B18] Glass N. L. Donaldson G. C. (1995). Development of primer sets designed for use with the PCR to amplify conserved genes from filamentous ascomycetes. *Appl. Environ. Microbiol.* 61 1323–1330. 10.1128/aem.61.4.1323-1330.1995 7747954 PMC167388

[B19] Gordon T. R. (2017). *Fusarium oxysporum* and the *Fusarium wilt* syndrome. *Annu. Rev. Phytopathol.* 55 23–39. 10.1146/annurev-phyto-080615-095919 28489498

[B20] Huang X. Q. Cai Z. C. (2017). Soil microbes and control of soil-borne diseases. *Bull. Chin. Acad. Sci.* 32 593–600.

[B21] Jiang C. H. Yao X. F. Mi D. D. Li Z. J. Yang B. Y. Zheng Y. (2019). Comparative transcriptome analysis reveals the biocontrol mechanism of *Bacillus velezensis* F21 against *Fusarium wilt* on watermelon. *Front. Microbiol.* 10:652. 10.3389/fmicb.2019.00652 31001229 PMC6456681

[B22] Karimi K. Amini J. Harighi B. Bahramnejad B. (2012). Evaluation of biocontrol potential of *Pseudomonas* and *Bacillus* spp. against *Fusarium wilt* of chickpea. *Aust. J. Crop Sci.* 6 695–703.

[B23] Kashiwa T. Kozaki T. Ishii K. Turgeon B. G. Teraoka T. Komatsu K. (2017). Sequencing of individual chromosomes of plant pathogenic *Fusarium oxysporum*. *Fungal Genet. Biol.* 98 46–51. 10.1016/j.fgb.2016.12.001 27919652

[B24] Kaur R. Kaur J. Singh R. (2010). Nonpathogenic *Fusarium* as a biological control agent. *Plant Pathol. J.* 61 1213–1217. 10.3923/ppj.2010.79.91

[B25] Kim J. D. Han J. W. Lee S. C. Lee D. Kim B. S. (2011). Disease control effect of strevertenes produced by *Streptomyces psammoticus* against tomato *Fusarium wilt*. *J. Agric. Food Chem.* 59 1893–1899. 10.1021/jf1038585 21314121

[B26] Kim M. J. Shim C. K. Kim Y. K. Hong S. J. Park J. H. Han E. J. (2017). Enhancement of seed dehiscence by seed treatment with *Talaromyces flavus* GG01 and GG04 in ginseng (*Panax ginseng*). *Plant Pathol. J.* 33 1–8. 10.5423/PPJ.OA.06.2016.0146 28167883 PMC5291393

[B27] Leidich S. D. Drapp D. A. Orlean P. (1994). A conditionally lethal yeast mutant blocked at the first step in glycosyl phosphatidylinositol anchor synthesis. *J. Biol. Chem.* 269 10193–10196. 10.1016/S0021-9258(17)34042-5 8144596

[B28] Levin D. E. (2011). Regulation of cell wall biogenesis in *Saccharomyces cerevisiae*: the cell wall integrity signaling pathway. *Genetics* 189 1145–1175. 10.1534/genetics.111.128264 22174182 PMC3241422

[B29] Li N. Alfiky A. Wang W. Islam M. Nourollahi K. Liu X. (2018). Volatile compound-mediated recognition and inhibition between *Trichoderma* biocontrol agents and *Fusarium oxysporum*. *Front. Microbiol.* 9:2614. 10.3389/fmicb.2018.02614 30455673 PMC6231246

[B30] López-Fernández L. Ruiz-Roldán C. Pareja-Jaime Y. Prieto A. Khraiwesh H. Roncero M. I. (2013). The *Fusarium oxysporum* gnt2, encoding a putative N-acetylglucosaminetransferase, is involved in cell wall architecture and virulence. *PLoS One* 8:e84690. 10.1371/journal.pone.0084690 24416097 PMC3886883

[B31] Mardones W. Callegari E. Eyzaguirre J. (2018a). Corncob and sugar beet pulp induce specific sets of lignocellulolytic enzymes in *Penicillium purpurogenum*. *Mycology* 10 118–125. 10.1080/21501203.2018.1517830 31069125 PMC6493289

[B32] Mardones W. Di Genova A. Cortés M. P. Travisany D. Maass A. Eyzaguirre J. (2018b). The genome sequence of the soft-rot fungus *Penicillium purpurogenum* reveals a high gene dosage for lignocellulolytic enzymes. *Mycology* 9 59–69. 10.1080/21501203.2017.1419995 30123662 PMC6059080

[B33] McKeen C. D. Wensley R. N. (1961). Longevity of *Fusarium oxysporum* in soil tube culture. *Science* 134 1528–1529. 10.1126/science.134.3489.1528 17800128

[B34] Orlean P. (2012). Architecture and biosynthesis of the *Saccharomyces cerevisiae* cell wall. *Genetics* 192 775–818. 10.1534/genetics.112.144485 23135325 PMC3522159

[B35] Pittet M. Conzelmann A. (2007). Biosynthesis and function of GPI proteins in the yeast *Saccharomyces cerevisiae*. *BBA Mol. Cell Biol. Lipids* 1771 405–420. 10.1016/j.bbalip.2006.05.015 16859984

[B36] Postma J. Scheper R. Schilder M. T. (2010). Effect of successive cauliflower plantings and *Rhizoctonia solani* AG 2-1 inoculations on disease suppressiveness of a suppressive and a conducive soil. *Soil Biol. Biochem.* 42 804–812. 10.1016/j.soilbio.2010.01.017

[B37] Schlatter D. Kinkel L. Thomashow L. Weller D. Paulitz T. (2017). Disease suppressive soils: new insights from the soil microbiome. *Phytopathology* 107 1284–1297. 10.1094/PHYTO-03-17-0111-RVW 28650266

[B38] Shen Z. Z. Penton C. R. Lv N. N. Xue C. Yuan X. F. Ruan Y. Z. (2017). Banana *Fusarium wilt* disease incidence is influenced by shifts of soil microbial communities under different monoculture spans. *Microb. Ecol.* 75 739–750. 10.1007/s00248-017-1052-5 28791467

[B39] Shi L. Du N. Shu S. Sun J. Li S. Guo S. (2017). *Paenibacillus polymyxa* NSY50 suppresses *Fusarium wilt* in cucumbers by regulating the rhizospheric microbial community. *Sci. Rep.* 7:41234. 10.1038/srep41234 28198807 PMC5304210

[B40] Spink D. S. Rowe R. C. (1989). Evaluation of *Talaromyces flavus* as a biological control agent against *Verticillium dahliae* in potato. *Plant Dis.* 73 230–236. 10.1094/PD-73-0230

[B41] Srinivas C. Nirmala Devi D. Narasimha Murthy K. Mohan C. D. Lakshmeesha T. R. Singh B. (2019). *Fusarium oxysporum* f. sp. *lycopersici* causal agent of vascular wilt disease of tomato: biology to diversity-a review. *Saudi J. Biol. Sci.* 26 1315–1324. 10.1016/j.sjbs.2019.06.002 31762590 PMC6864208

[B42] Strub C. Dieye C. A. T. Nguyen P. A. Constancias F. Durand N. Guendouz S. (2019). Transcriptomes of the interaction between *Fusarium verticillioides* and a *Streptomyces* strain reveal the fungal defense strategy under the pressure of a potential biocontrol agent. *Fungal Biol.* 125 78–88. 10.1016/j.funbio.2019.11.007 33518208

[B43] Su L. Niu Y. C. (2018). Multilocus phylogenetic analysis of *Talaromyces* species isolated from cucurbit plants in China and description of two new species, *T. cucurbitiradicus* and *T. endophyticus*. *Mycologia* 110 375–386. 10.1080/00275514.2018.1432221 29737936

[B44] Tang L. Yu X. Zhang L. Zhang L. Chen L. Zou S. (2020). Mitochondrial FgEch1 is responsible for conidiation and full virulence in *Fusarium graminearum*. *Curr. Genet.* 66 361–371. 10.1007/s00294-019-01028-z 31463774

[B45] Tao C. Y. Li R. Xiong W. Shen Z. Z. Liu S. S. Wang B. B. (2020). Bio-organic fertilizers stimulate indigenous soil *Pseudomonas* populations to enhance plant disease suppression. *Microbiome* 8:137. 10.1186/s40168-020-00892-z 32962766 PMC7510105

[B46] Thangavelu R. Gopi M. (2015). Combined application of native *Trichoderma* isolates possessing multiple functions for the control of *Fusarium wilt* disease in banana cv. Grand Naine. *Biocontrol Sci. Technol.* 25 1147–1164. 10.1080/09583157.2015.1036727

[B47] Ueki A. Takehara T. Ishioka G. Kaku N. Ueki K. (2017). Degradation of the fungal cell wall by clostridial strains isolated from soil subjected to biological soil disinfestation and biocontrol of *Fusarium wilt* disease of spinach. *Appl. Microbiol. Biotechnol.* 101 8267–8277. 10.1007/s00253-017-8543-7 28967048

[B48] Varriale S. Houbraken J. Granchi Z. Pepe O. Cerullo G. Ventorino V. (2018). *Talaromyces borbonicus*, sp. nov., a novel fungus from biodegraded *Arundo donax* with potential abilities in lignocellulose conversion. *Mycologia* 110 316–324. 10.1080/00275514.2018.1456835 29843575

[B49] Visagie C. M. Yilmaz N. Frisvad J. C. Houbraken J. Seifert K. A. Samson R. A. (2015). Five new *Talaromyces* species with ampulliform-like phialides and globose rough walled conidia resembling *T. verruculosus*. *Mycoscience* 56 486–502. 10.1016/j.myc.2015.02.005

[B50] Wang B. Wang L. (2013). *Penicillium kongii*, a new terverticillate species isolated from plant leaves in China. *Mycologia* 105 1547–1554. 10.3852/13-022 23921240

[B51] Wang Y. F. Tian Y. H. Wei Y. J. Gao K. X. (2019). Screening of optimum combination of biocontrol bacteria+hymexazol and studies on its control effect on bitter gourd *Fusarium wilt*. *China Vegetables* 7 67–73.

[B52] Wei Z. Hu J. Gu Y. A. Yin S. X. Xu Y. C. Jousset A. (2018). Ralstonia solanacearum pathogen disrupts bacterial rhizosphere microbiome during an invasion. *Soil Biol. Biochem.* 118 8–17. 10.1016/j.soilbio.2017.11.012

[B53] White T. J. Bruns T. Lee S. Taylor J. (1990). *Amplification and Direct Sequencing of Fungal Ribosomal RNA Genes for Phylogenetic.* New York, NY: Academic Press, 315–322. 10.1016/B978-0-12-372180-8.50042-1

[B54] Wu Q. Sun R. Ni M. Yu J. Li Y. Yu C. (2017). Identification of a novel fungus, *Trichoderma asperellum* GDFS1009, and comprehensive evaluation of its biocontrol efficacy. *PLoS One* 12:e0179957. 10.1371/journal.pone.0179957 28644879 PMC5482467

[B55] Xiao C. H. Yu X. M. He Y. Y. Deng X. Z. Tang E. (2008). Studies on the biological characteristics of the pathogen of cucurbit wilt. *Plant Prot.* 34 83–86.

[B56] Xue C. Penton C. R. Shen Z. Z. Zhang R. F. Huang Q. W. Li R. (2015). Manipulating the banana rhizosphere microbiome for biological control of Panama disease. *Sci. Rep.* 5:11124. 10.1038/srep14596 26242751 PMC4525139

[B57] Zhang B. G. Zhang J. Liu Y. Shi P. Wei G. H. (2018). Co-occurrence patterns of soybean rhizosphere microbiome at a continental scale. *Soil Biol. Biochem.* 118 178–186. 10.1016/j.soilbio.2017.12.011

[B58] Zhao S. Chen X. Deng S. Dong X. Song A. Yao J. (2016). The effects of fungicide, soil fumigant, bio-organic fertilizer and their combined application on chrysanthemum *Fusarium wilt* controlling, soil enzyme activities and microbial properties. *Molecules* 21:526. 10.3390/molecules21040526 27110753 PMC6273536

[B59] Zhu W. Wei W. Wu Y. Zhou Y. Peng F. Zhang S. (2017). BcCFEM1, a CFEM domain-containing protein with putative GPI-anchored site, is involved in pathogenicity, conidial production, and stress tolerance in Botrytis cinerea. *Front. Microbiol.* 8:1807. 10.3389/fmicb.2017.01807 28979251 PMC5611420

