# Supplementary material for: Isolation and Identification of Talaromyces sp. Strain Q2 and Its Biocontrol Mechanisms Involved in the Control of Fusarium Wilt
Source: Front Microbiol. 2021 Oct 8;12:724842. doi: 10.3389/fmicb.2021.724842 (PMC8531730; doi:10.3389/fmicb.2021.724842)
Supplement: Supplementary Figure 1 — Influence of different nutrients and culture conditions on TpQ2 colony growth. [file Presentation_1.pdf]

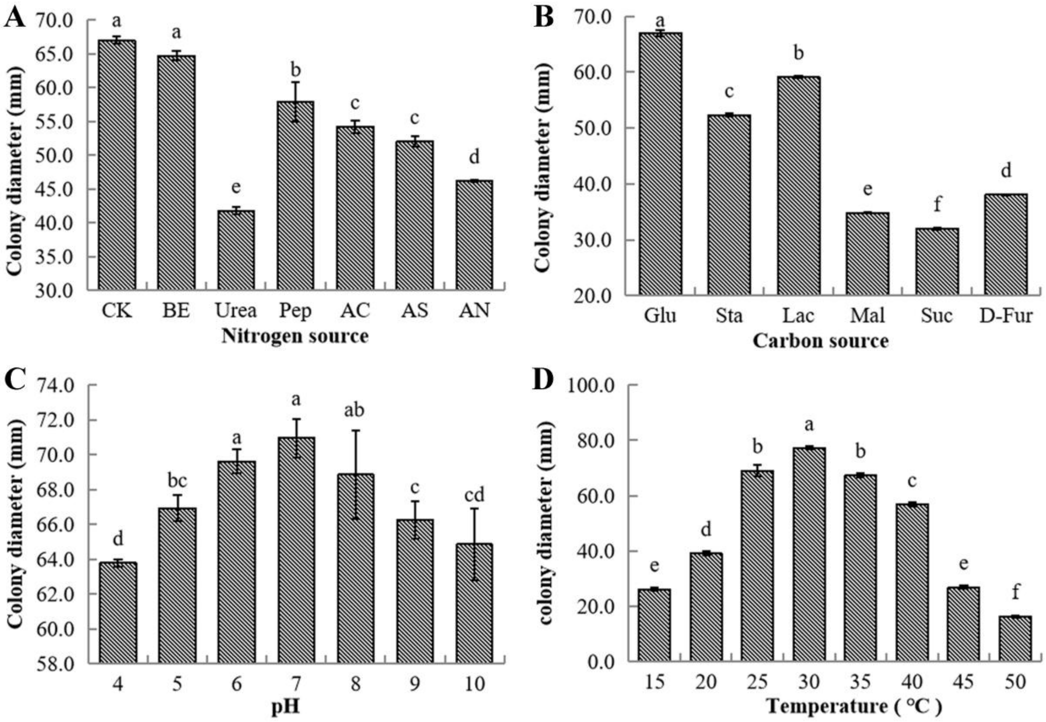

Fig. S1 Influence of different nutrients and culture conditions on TpQ2 colony growth.

Glu, Glucose; Sta, Starch; Lac, Lactose; Mal, Maltose; Suc, Sucrose; D-Fur, D-Furctose; CK, control; BE, Beef Extract; Urea, Carbamide; Pep, Peptone; AC, Ammonium chloride; AS, Ammonium sulfate; AN, Ammonium nitrate. Different letters in the same column indicate significant statistical difference at *P*<0.05 level determined using the Student-Newman-Keuls test, the below is same.

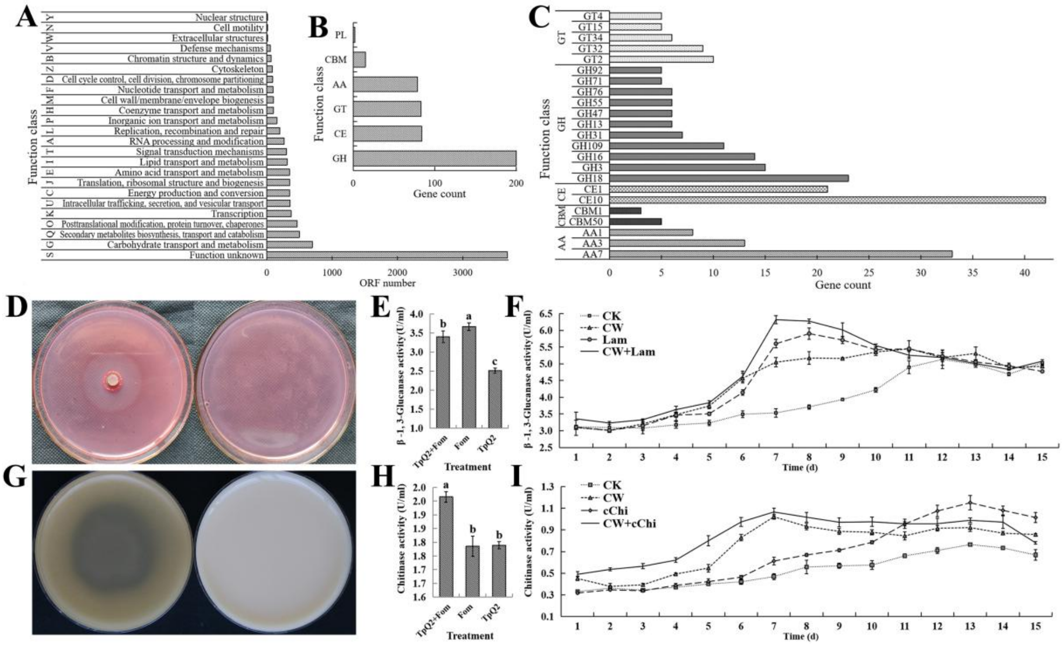

Fig. S2 Genome-wide identification and the potential function analysis of the beneficial TpQ2

A: Functional classification of genes identified in TpQ2 was performed using eggNOG (evolutionary genealogy of genes: Non-supervised Orthologous Groups). B: Types of CAZymes found in TpQ2. GH, glycoside Hydrolases; CE, carbohydrate esterases; GT, glycosyl transferases; AA, auxiliary Activities; CBM, carbohydrate-binding modules; PL, polysaccharide Lyases. C: Abundant CAZymes found in TpQ2. D: Activity of β-1, 3-glucanase in TpQ2 grown on the agar plates containing laminarin. E: The levels of β-1, 3-glucanase of culture filtrate in the pure culture or co-culture of TpQ2 and Fom. F: The levels of β-1, 3-glucanase produced by TpQ2 grown on different inducing substrates. G: Activity of chitinase in TpQ2 grown on the agar plates containing colloidal chitin. H: The levels of chitinase of culture filtrate in the pure culture or co-culture of TpQ2 and Fom. I: The levels of chitinase produced by TpQ2 grown on different inducing substrates. cChi, colloidal chitin; Lam, laminarin; CW, the filamentous fragments of Fom; CK (control treatment), TpQ2 in PDB liquid medium; CW, TpQ2 in PDB liquid medium + 2mg·mL^{-1} CW; cChi, TpQ2 in PDB liquid medium + 0.1mg·mL^{-1} cChi; CW+cChi, TpQ2 in PDB liquid medium + 2mg·mL^{-1} CW + 0.1mg·mL^{-1} cChi; Lam: TpQ2 in PDB liquid medium + 0.1mg·mL^{-1} Lam; CW+Lam, TpQ2 in PDB liquid medium + 2mg·mL^{-1} CW + 0.1mg·mL^{-1} Lam. Culture condition was 7 days at 28℃ with 180 rpm shaking.

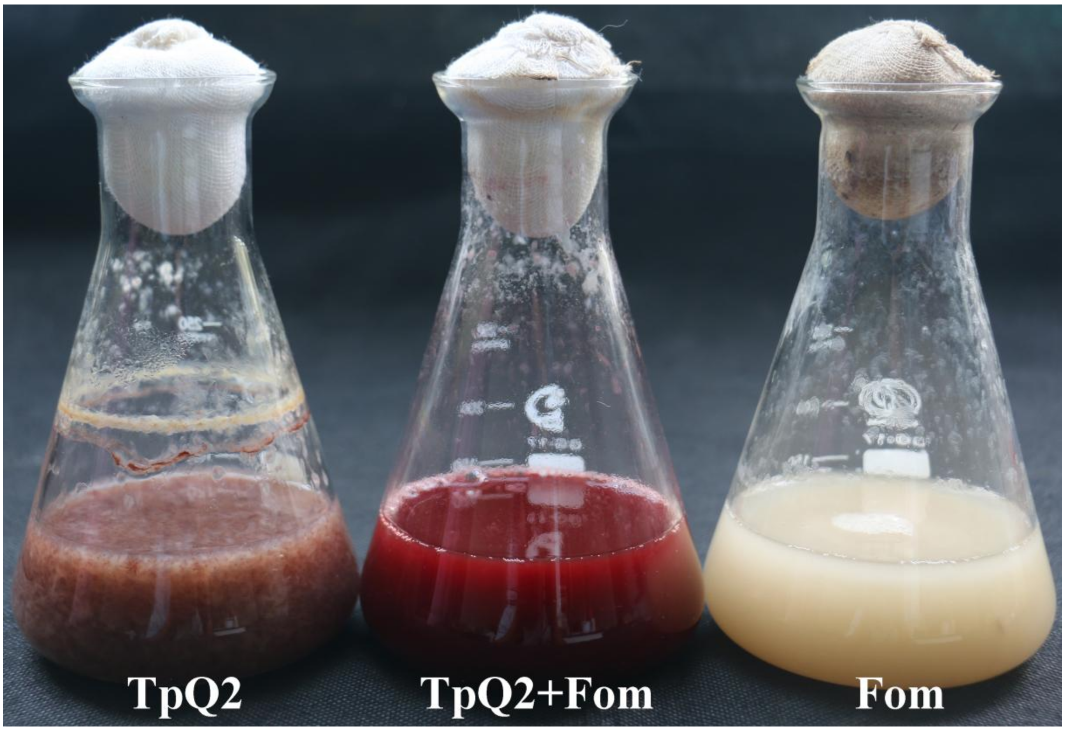

Fig. S3 Single and co-culture of TpQ2 and Fom.

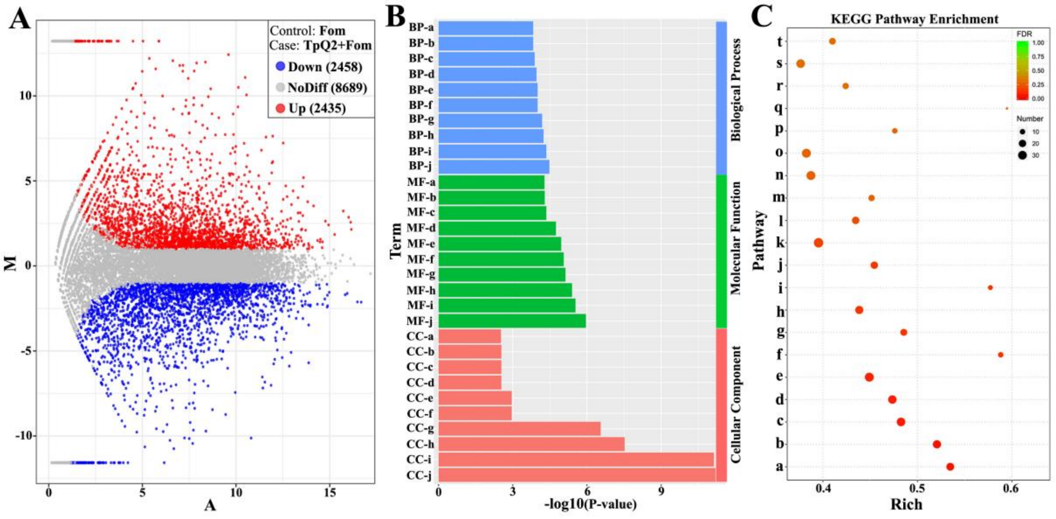

Fig. S4 MA map analysis (A), Gene Ontology analysis (B), and KEGG enrichment analysis (C) using DEGs identified between the Fom and TpQ2+Fom treatments.

BP-a, carboxylic acid transport(GO:0046942); BP-b, organic acid transport (GO:0015849); BP-c, ion transport (GO:0006811); BP-d, oxidation−reduction process (GO:0055114); BP-e, carboxylic acid transmembrane transport (GO:1905039); BP-f, organic acid transmembrane transport (GO:1903825); BP-g, anion transport (GO:0006820); BP-h, amino acid transmembrane transport (GO:0003333); BP-I, anion transmembrane transport (GO:0098656); BP-j, transmembrane transport (GO:0055085); CC-a, eukaryotic 43S preinitiation complex (GO:0016282); CC-b, translation preinitiation complex (GO:0070993); CC-c, eukaryotic 48S preinitiation complex (GO:0033290); CC-d, eukaryotic translation initiation factor 3 complex (GO:0005852); CC-e, intrinsic component of plasma membrane (GO:0031226); CC-f, integral component of plasma membrane (GO:0005887); CC-g, membrane (GO:0016020); CC-h, membrane part (GO:0044425); CC-i, integral component of membrane (GO:0016021); CC-j, intrinsic component of membrane (GO:0031224); MF-a, oxidoreductase activity, acting on a sulfur group (GO:0016667); MF-b, substrate−specific transporter activity (GO:0022892); MF-c, disulfide oxidoreductase activity (GO:0015036); MF-d, RNA polymerase II transcription factor activity, s (GO:0000981); MF-e, anion transmembrane transporter activity (GO:0008509); MF-f, transporter activity (GO:0005215); MF-g, ion transmembrane transporter activity (GO:0015075); MF-h, substrate−specific transmembrane transporter activity (GO:0022891); MF-i, transmembrane transporter activity (GO:0022857); MF-j, oxidoreductase activity (GO:0016491); a, glyoxylate and dicarboxylate metabolism; b, glutathione metabolism; c, valine, leucine and isoleucine degradation; d, glycerophospholipid metabolism; e, phenylalanine metabolism; f, nitrogen metabolism; g, propanoate

metabolism; h, pyruvate metabolism; i, ABC transporters; j, galactose metabolism; k, tyrosine metabolism; l, glycerolipid metabolism; m, butanoate metabolism; n, arginine and proline metabolism; o, starch and sucrose metabolism; p, nicotinate and nicotinamide metabolism; q, arachidonic acid metabolism; r, methane metabolism; s, amino sugar and nucleotide sugar metabolism; t, pentose phosphate pathway.

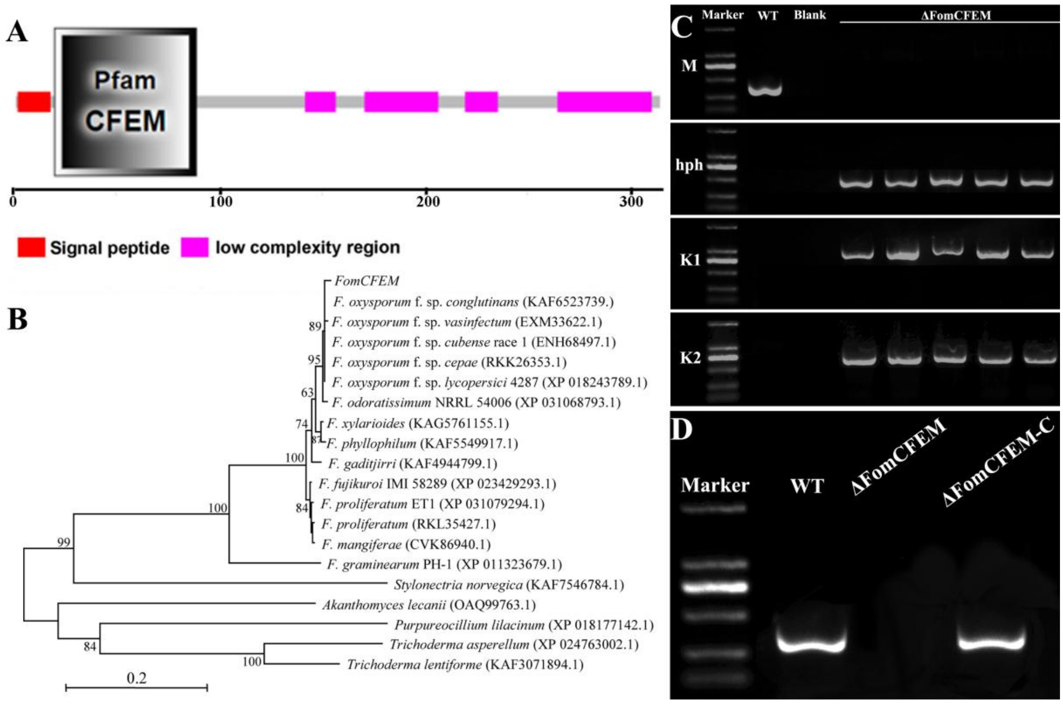

Fig. S5 Domains predicted in *FomCFEM*, phylogenetic tree, and PCR screen of Fom *FomCFEM* deletion and complementation mutants.

A: A schematic illustration of *FomCFEM*. The domains in *FomCFEM* was predicted using the SMART software. B: A phylogenetic tree showing the relationships between *FomCFEM* and other proteins with a CFEM domain in other fungi. C: The *FomCFEM* deletion mutant was made through transformation of the wild type Fom with the construct carrying a hygromycin resistance (*hph*) gene flanked by an upstream and a downstream sequence of *FomCFEM*. D: PCR screen of *FomCFEM* deletion (*ΔFomCFEM*) and complementation mutants (*ΔFomCFEM-C*)

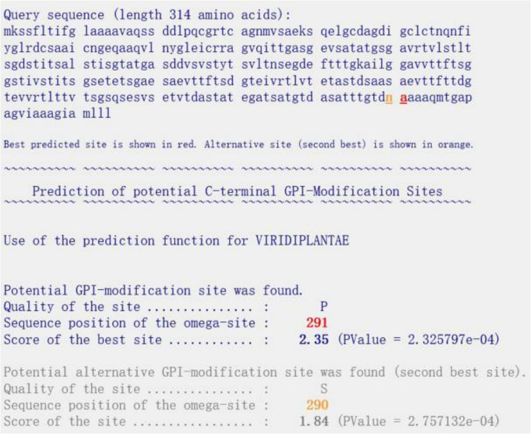
```
Query sequence (length 314 amino acids):
mkssfltifg laaaavaqss ddlpqcgrtc agnmvsaeks qelgcdagdi gclctnqnfi
yglrdcsaai cngeqaaqvl nygleicrra gvqittgasg evsatatgsg avrtvlstlt
sgdstitsal stisgtatga sddvsvstyt svltnsegde ftttgkailg gavvttftsg
gstivstits gsetetsgae saevttftsd gteivrtlvt etastdsaas aevttfttdg
tevvrtlttv tsgsqsesvs etvtdastat egatsatgtd asatttgtdn aaaaqmtgap
agviaaagia mlll

Best predicted site is shown in red. Alternative site (second best) is shown in orange.

~~~~~~~~~~ ~~~~~~~~~~ ~~~~~~~~~~ ~~~~~~~~~~ ~~~~~~~~~~ ~~~~~~~~~~
    Prediction of potential C-terminal GPI-Modification Sites
~~~~~~~~~~ ~~~~~~~~~~ ~~~~~~~~~~ ~~~~~~~~~~ ~~~~~~~~~~ ~~~~~~~~~~

Use of the prediction function for VIRIDIPLANTAE


Potential GPI-modification site was found.
Quality of the site ............... :        P
Sequence position of the omega-site :      291
Score of the best site ............ :     2.35 (PValue = 2.325797e-04)

Potential alternative GPI-modification site was found (second best site).
Quality of the site ............... :        S
Sequence position of the omega-site :      290
Score of the site ................. :     1.84 (PValue = 2.757132e-04)
```

Fig. S6 Prediction of GPI site in *FomCFEM*.

Tool in this website (http://mendel.imp.ac.at/gpi/plant_server.html) was used to predict the potential GPI modification site in *FomCFEM*.

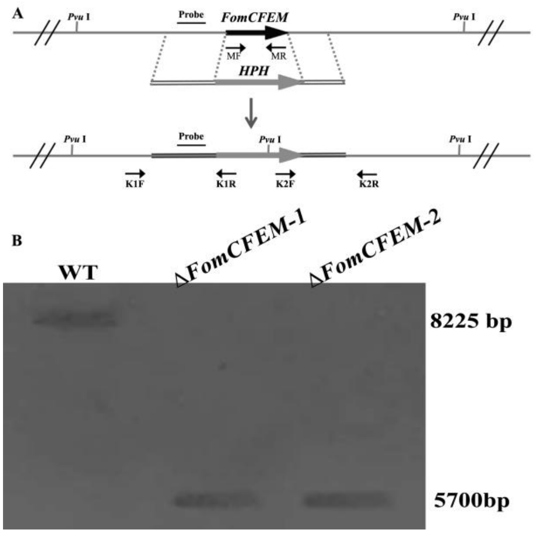

Fig. S7 A schematic diagram showing detections of *FomCEFM* deletion mutants

A: Strategy used to produce Δ*FomCEFM* mutant construct. B: Southern blot analysis of two candidate Δ*FomCEFM* deletion mutants.

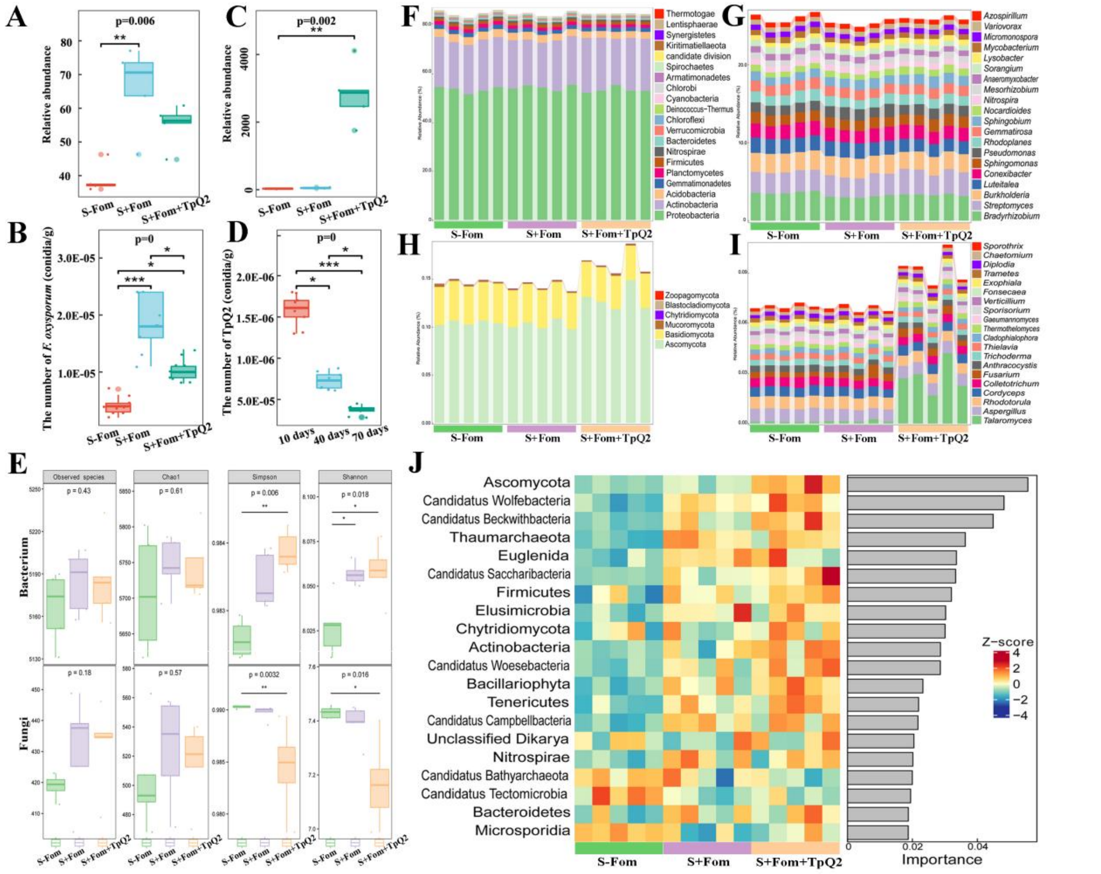

Fig. S8 Influences of TpQ2 on microbial alpha diversity index and community composition

A: Relative abundances of *F. oxysporum* found in the S-Fom, S+Fom, and S+Fom+TpQ2 samples. B: Numbers of *F. oxysporum* isolated from the S-Fom, S+Fom, and S+Fom+TpQ2 samples cultured on the Komada's medium. C: Relative abundance of TpQ2 was determined through comparison of TpQ2 genome to the entire metagenome sequencing data. D: The ability of TpQ2 to colonize soil at various days post inoculation. E: Microbial alpha diversity index was determined using the metagenome sequencing data from the S-Fom, S+Fom, and S+Fom+TpQ2 samples. F: Relative abundance of bacterial taxa found in the S-Fom, S+Fom, and S+Fom+TpQ2 samples at the phylum level. G: Relative abundance of bacterial taxa found in the S-Fom, S+Fom, and S+Fom+TpQ2 samples at the genus level. H: Relative abundance of fungal taxa found in the S-Fom, S+Fom, and S+Fom+TpQ2 samples at the phylum level. I: Relative abundance of fungal taxa found in the S-Fom, S+Fom, and S+Fom+TpQ2 samples at the genus level. J: The top 20 biomarker microbial phylum were identified by applying Random Forests regression of their relative abundances in soil against the colonization of Fom and TpQ2.

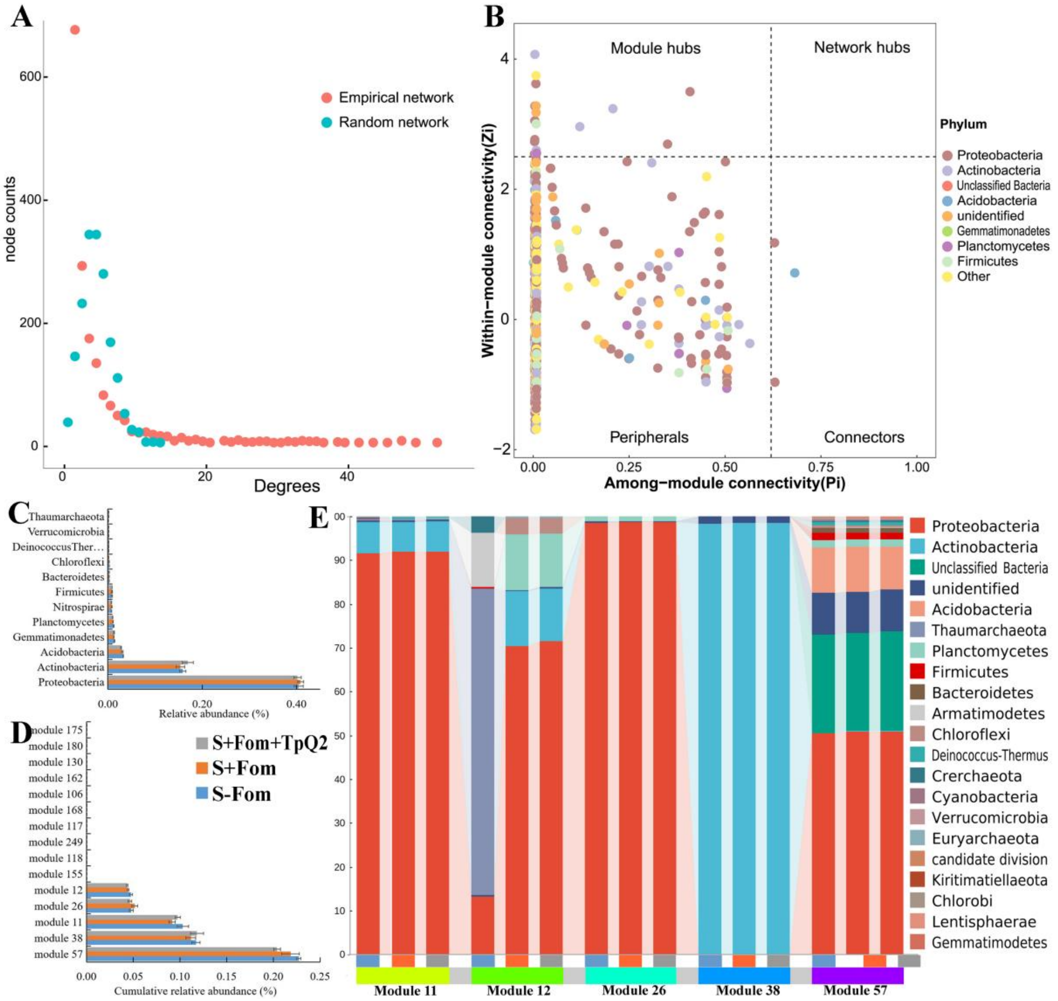

Fig. S9 Network level microbial features in the co-occurrence networks

A: Distributions of degrees. B: Zi-Pi plot nodes. C: Relative abundances of main microbial phylum in the co-occurrence networks. D: Cumulative relative abundances of all bacteria and fungi in the main modules of the co-occurrence networks. E: Microbial compositions in the modules at the phylum level.

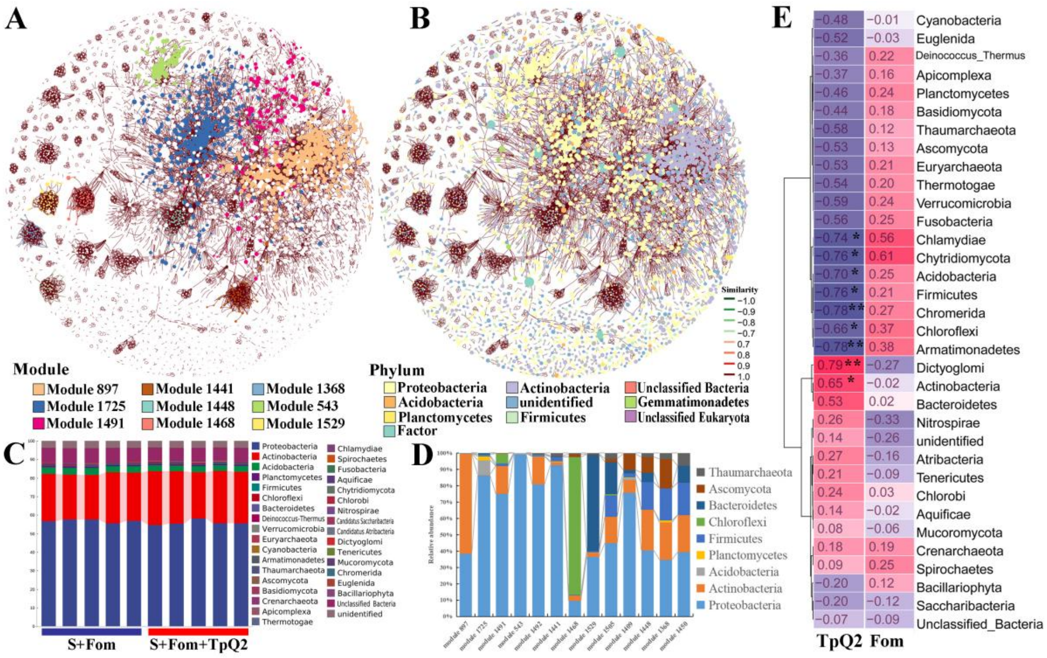

Fig. S10 Co-occurrence networks of soil microbial communities

A: Co-occurrence networks was constructed using the data set from the S+Fom and S+Fom+TpQ2 samples using the correlation analysis to reveal the associations among the modules. B: Compositions of the co-occurrence networks at the phylum-level. C: Microbial compositions of the 13 bigger modules at the phylum level. D: Key microbes in the modules. E: Spearman's correlation analysis of variations of key microbes after co-culture of Fom and TpQ2.
